# UBA1-depleted neutrophils disrupt immune homeostasis and induce VEXAS-like autoinflammatory disease in mice

**DOI:** 10.1172/JCI193011

**Published:** 2025-09-04

**Authors:** Ge Dong, Jingjing Liu, Wenyan Jin, Hongxi Zhou, Yuchen Wen, Zhiqin Wang, Keyao Xia, Jianlin Zhang, Linxiang Ma, Yunxi Ma, Lorie Chen Cai, Qiufan Zhou, Huaquan Wang, Wei Wei, Ying Fu, Zhigang Cai

**Affiliations:** 1 Tianjin Key Laboratory of Inflammatory Biology, Department of Pharmacology, School of Basic Medical Science,; 2 State Key Laboratory of Experimental Hematology,; 3 The Province and Ministry Co-sponsored Collaborative Innovation Center for Medical Epigenetics, School of Basic Medical Science, and; 4 Department of Bioinformatics, School of Basic Medical Science, Tianjin Medical University, Tianjin, China.; 5 Department of Hematology, Tianjin Medical University, Tianjin General Hospital, Tianjin, China.; 6 Department of Rheumatology and Immunology, Tianjin Medical University Tianjin General Hospital, Tianjin, China.; 7 Department of Neurology and Institute of Neurology of First Affiliated Hospital, Institute of Neuroscience, and Fujian Key Laboratory of Molecular Neurology, Fujian Medical University, Fuzhou, China.

**Keywords:** Hematology, Inflammation, Hematopoietic stem cells, Neutrophils, Ubiquitin-proteosome system

## Abstract

Vacuoles, E1 enzyme, X-linked, autoinflammatory, somatic (VEXAS) syndrome is a hemato-rheumatoid disease caused by somatic *UBA1* mutations in hematopoietic stem cells (HSCs). The pathogenic cell type(s) responsible for the syndrome are unknown, and murine models recapitulating the disease are lacking. We report that loss of *Uba1* in various mouse hematopoietic cell types resulted in pleiotropic consequences and demonstrate that an approximate 70% loss of *Uba1* in neutrophils (NEs) of murine mutants induced nonlethal VEXAS-like symptoms. Depletion of *Uba1* in HSCs induced extensive hematopoietic cell loss, whereas depletion of *Uba1* in B cells, T cells, or megakaryocytes induced corresponding cell death, but these mutant mice appeared normal. Depletion of *Uba1* in monocytes and NEs failed to induce cell death, and the mutant mice were viable. Among the tested models, only depletion of *Uba1* in NEs induced autoinflammatory symptoms including increased counts and percentages of NEs, increased proinflammatory cytokines, presence of vacuoles in myeloid cells, splenomegaly, and dermatitis. Residual Uba1 was approximately 30% in the mutant NEs, which disrupted cellular hemostasis. Finally, genetic loss of the myeloid prosurvival regulator *Morrbid* partially mitigated the VEXAS-like symptoms. The established VEXAS-like murine model will further our understanding and treatment of the newly identified autoinflammatory syndrome prevalent among aged men.

## Abstract 2

Neutrophil loss of Uba1 results in VEXAS-like symptoms

## Introduction

It has been recognized for almost 40 years that protein ubiquitylation (also called ubiquitination), a posttranslational process that transfers the 76 amino acid peptide ubiquitin to host proteins in the nucleus or cytoplasm, is critical for nearly all aspects of eukaryotic biology and cellular homeostasis (1). Aberrant protein ubiquitylation induces cellular dysfunction in many aspects of cell activities for neuronal cells or immune cells, among other important cells, and may indicate disease (2). For example, among multiple mechanisms of ubiquitin signaling, ubiquitylation of histone in the nucleus regulates genome stability and gene expression (3), and ubiquitin activation in the cytoplasm is the first step of the E1-E2-E3 enzymatic cascade, which is critical for almost all proteins inside eukaryotic cells and maintains cellular homeostasis (4). Deficient protein ubiquitylation results in inadequate protein degradation, which in turn will generate overloaded proteins inside the cell and induce the unfolded protein stress response (UPR) (5, 6).

In the E1-E2-E3 enzymatic cascade, the functions of the enzymes E2 (~40 members) and E3 (>600 members) have been broadly studied, given that they may regulate only a specific or single aspect of physiology, and loss of 1 of the E2 and E3 members is insufficient to induce cell death (7, 8). However, given the limited number of E1 members (only 2 members in mice and humans: UBA1 and UBA6) and the lethality induced by loss of either UBA1 or UBA6, their function and action mechanisms in the in vivo model have not been well documented (9, 10). Interestingly, *UBA6* encodes only a cytoplasm isoform, UBA6, while *UBA1* encodes 2 isoforms, UBA1a and UBA1b. The UBA1a isoform is with nucleic signal and is located in the nucleus, whereas the UBA1b isoform protein is shorter in length and is located in the cytoplasm (11). In conditional knockout (CKO) models, the role of *Uba6* in mouse has been implicated in neuronal cells (12). The regulation of polyalanine stretch by UBA6 is suggested in the critical biological process (13). However, the in vivo role of UBA1 in mammals, including the respective roles of UBA1a and UBA1b isoforms, remains largely unknown (14).

Although complete loss of *UBA1* or *UBA6* is hypothesized to induce cell death, somatic mutations in these 2 genes in clinical samples offer alternative approaches to understanding their functions in eukaryotic cells. It has been reported that a germline mutation in *UBA1* (i.e., p.Met539Ile or p.Ser547Gly) induces X-linked infantile spinal muscular atrophy, a dysfunction of the nervous system (15, 16). Additionally, *UBA1^M41L^* mutation–induced loss of function of UBA1b isoform (p.Met41Leu) was implicated in a newly described autoinflammatory rheumatoid disease with hematologic complications, the vacuoles, E1 enzyme, X-linked, autoinflammatory, somatic (VEXAS) syndrome (17). The *UBA1^M41L^* mutation results in removal of the cytoplasm isoform UBA1b, while the nucleic isoform UBA1a is unchanged. The VEXAS syndrome occurs primarily in adult men, with rare reports of the syndrome in women, suggesting it is induced by loss of function of *UBA1* (18). Approximately 200 follow-up, retrospective clinical studies describing approximately 500 patients with VEXAS syndrome further revealed that various mutations in *UBA1* are associated with this severe adult-onset disease affecting organs including the blood, blood vessels, nose, ears, skin, lungs, and brain (18–33). In the first VEXAS report, loss of *UBA1b*, rather than *UBA1a*, homologs in zebrafish by gene-editing was described to induce systemic inflammation; however, the fish mutants were lethal and minimally reminiscent of the VEXAS symptoms in humans (17). Recently, through a cutting-edge gene editing strategy, a mouse cell line–based approach (in vitro cell culture) and a human hematopoietic stem cell–based (HSC-based) approach (hosted by chimeric mice) for modeling VEXAS was respectively reported by Chiaramida et al. (34) and by Molteni et al. in a meeting abstract (35). Although these 2 models are important and complementary for understanding the pathology of VEXAS, a genetically reliable and easy-setup in vivo VEXAS-like animal model that could be maintained in adult-aged animals is still lacking.

To define the major responsible cell type(s) and recapitulate human VEXAS in mammals, we aimed here to generate murine models by genetically manipulating the *Uba1* locus and to further explore the pathology and treatment of this autoinflammatory disease. We constructed various CKO models to dissect the discrete roles of *Uba1* in each one of the major hematopoietic cell types including, HSCs, lymphoid cells, megakaryocytes, and myeloid cells. Our study demonstrates that loss of *Uba1* in neutrophils (NEs) was critical for inducing VEXAS-like disorders and offers translational potential, suggesting that the VEXAS symptom in humans is likely attributed to a combinational hematological and immunological consequence of *UBA1* point mutation over a long-term journey of clonal hematopoiesis. Thus, complimentary to studies merely using clinical samples, our study suggests that mutant NEs are aberrant and equally as important as the mutant HSCs and play a critical role in driving autoinflammatory disease. In addition, our study suggests that treatment with IL-1b and IL-1R1 inhibitors (canakinumab or anakinra) or genetic loss of the myeloid prosurvival regulator *Morrbid* partially mitigated the VEXAS-like autoinflammatory symptoms in mutant mice.

## Results

### A Cre/fl approach to model VEXAS syndrome.

**Figure 1:**
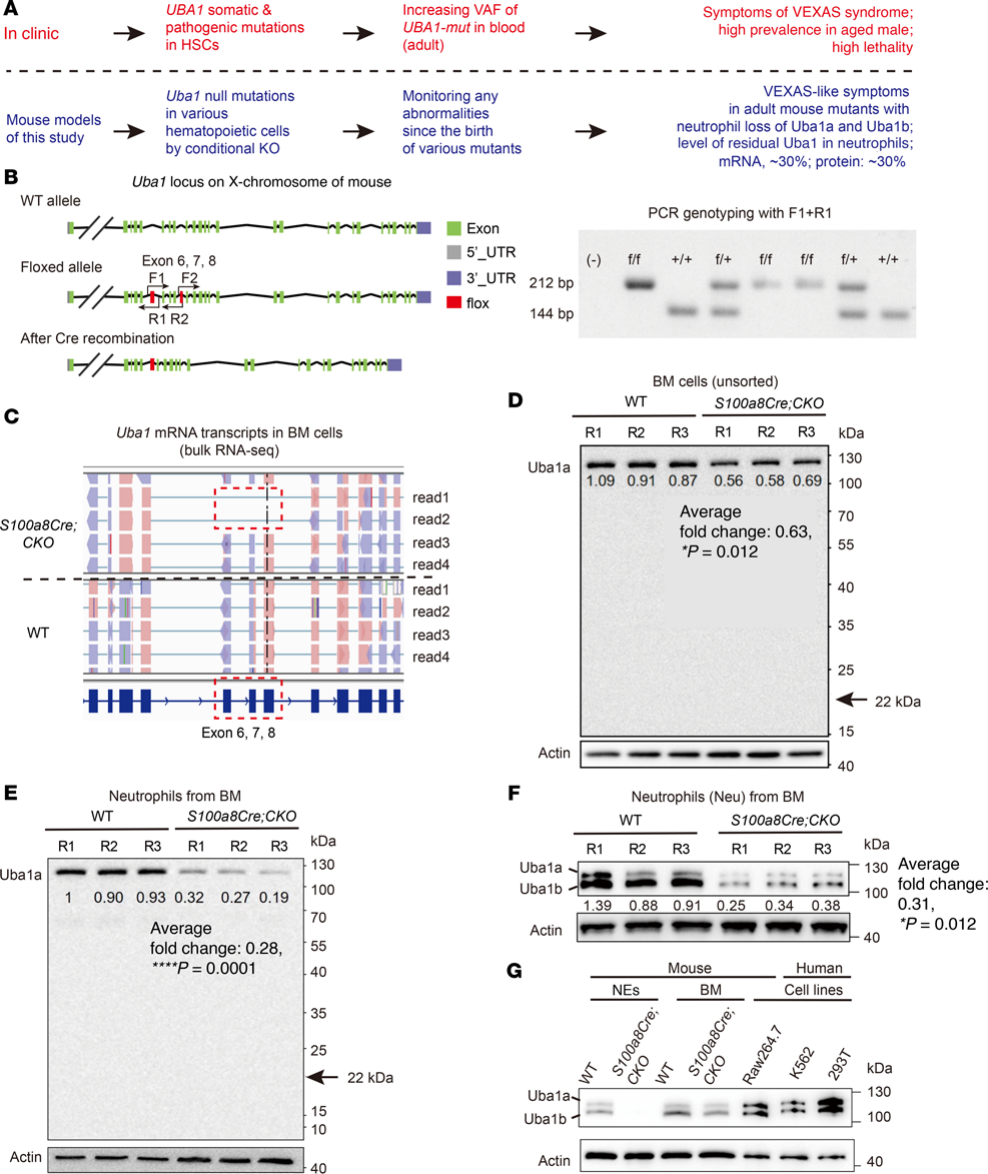
Conditional depletion of Uba1 in mice. (**A**) A brief introduction of VEXAS syndrome occurrence in the clinic and in the present study using CKO mouse models. VAF, variant allele fraction. (**B**) Gene structure of *Uba1* in mice and the CKO strategy in the present study. The mouse *Uba1* locus is in the X-chromosome as human *UBA1*. Left panel: Gene structure of *Uba1* in the WT allele and the fl knock-in allele. Right panel: The primers F1/R1 were used to distinguish the WT and fl alleles in the PCR genotyping. (**C**) Disruption of *Uba1* transcripts was revealed by sequencing read alignment in the bulk RNA-Seq BM cell datasets. The *S100a8Cre*-CKO mice were used here for transcriptional chimerism analysis. (**D**) Expression of Uba1 protein in total BM cells by an antibody specific to the Uba1a isoform. Note that no truncated Uba1a protein was detected (arrow marks the predicted molecular weight of the putative truncated isoform, 22 kDa). Three biological repeats (R1, R2, and R3) were used for WT and CKO mice. (**E**) Expression of the Uba1 isoform in purified NEs by the Uba1a-specific antibody. Note that the residual level of Uba1a in NEs isolated from *S100a8Cre*-CKO BM was approximately 28% of that in WT controls (*P* = 0.0001). Again, no truncated Uba1a protein was detected (arrow, 22 kDa). (**F**) NE depletion of Uba1a and Uba1b isoforms determined by an alternative antibody sensitive to the 2 Uba1 isoforms. Note that the residual level of Uba1 (2 isoforms in total) in *S100a8Cre*-CKO BM was approximately 31% of that in WT controls (*P* = 0.012). (**G**) Expression of the 2 Uba1 isoforms (Uba1a and Uba1b) in primary BM cells of WT and *S100a8Cre*-CKO mice, in the mouse cell line Raw264.7, and in the 2 human cell lines K562 and 293T. *P < 0.05 and ****P < 0.0001, by Student’s *t*-test. *n* = 3~6 biological repeats. R1/R2/R3 indicates 3 independent biological repeats.

*UBA1* in humans and *Uba1* in mice are at the X-chromosome. The encoded proteins (UBA1 vs. Uba1) shares the same protein length and almost the same protein molecular weight (1,058 amino acids; for the full-length isoform, 117.849 kDa in humans vs. 117.808 kDa in mice). The identity of the protein sequences is as high as 95% and similarity is as high as 98% (Supplemental Figure 1A; supplemental material available online with this article; https://doi.org/10.1172/JCI193011DS1). Although VEXAS syndrome is induced by a spontaneous somatic mutation in HSC *UBA1* in humans (upper panel, Figure 1A), we report here the results of Cre/fl-based CKO models by observing the primary mutant mice or by transplantation of mutant bone marrow (BM) cells into chimeric mice. The outcomes suggest that the CKO strategy is technically feasible and that VEXAS syndrome is easy to model. In addition, the VEXAS-like mutant mice could be maintained during adult age, since we found that NE loss of *Uba1* (null mutation) induced VEXAS-like symptoms (lower panel, Figure 1A). Because of the unavailability of certain *Cre* strains, the present study does not cover the specific roles of *Uba1* for erythroid cells, natural killer cells, mast cells, eosinophils, or basophils, but other major cell types in hematopoiesis system have been covered and described in detail. Here, we report the results from the 9 different CKO mouse models covering deletion of *Uba1* in HSCs (3 lines in total), pan-lymphoid cells (2 lines in total; among them, 1 line for B cells and 1 line for T cells), and pan-myeloid cells (4 lines in total; among them, 1 line for megakaryocytes, 2 lines for monocytes/macrophages, and 1 line for NE).

As shown in the left panel of Figure 1B, we generated a floxed strain at the *Uba1* locus, where the exons 6–8 are removed when the *Uba1^fl^* mice were crossed with certain *Cre* lines. The floxed strain appeared normal, like WT male and female mice (for males, *Uba1^fl/y^* is normal as *Uba1^+/y^*; for females, *Uba1^fl/fl^* and *Uba1^fl/+^* are normal as *Uba1^+/+^*). PCR genotyping with F1 and R1 primers successfully distinguished the floxed allele from the WT allele (right panel, Figure 1B). As VEXAS syndrome is a disorder mostly affecting older men, we mainly generated male CKO mutant mice (mutant male mice, referred to hereafter as *Cre CKO* or *Cre Uba1^fl/y^* mice and WT male mice, referred to hereafter as *Uba1^fl/y^* mice) and collected related tissues for the subsequent studies.

As CKO mice generally manifest chimerism of WT and mutant mRNA transcripts of *Uba1* in certain tissues, we first verified the disruption of the *Uba1* transcripts by aligning the bulk sequencing reads in the pool of all reads to the mouse reference genome. As shown in Figure 1C, we observed that some sequencing reads indeed had no exons 6–8, which was expected with the Cre/fl gene-targeting strategy (the CKO mice used for mRNA transcript analysis were *S100a8Cre-*CKO; see below). Removal of Uba1 exons 6–8 by Cre resulted in 331 bp deletion and an early occurrence of a stop codon at exon 10 in the mRNA transcript. The putative truncated protein was predicted to be 200 amino acids in protein length and 22 kDa in protein molecular weight. Through the assays with the Uba1a isoform–specific antibody, we failed to identify a real truncated protein (indicated by the arrow in Figure 1, D and E) in total BM cells or in NEs from *S100a8Cre*-CKO mice. Furthermore, in total BM cells, we determined that the average level of the Uba1a isoform in the *S100a8Cre*-CKO mutant was 63% of that in WT controls (*P* = 0.012, Figure 1D). In the purified NEs, we determined that the average level of the Uba1a isoform in the *S100a8Cre*-CKO mutant was 28% of that in WT controls, suggesting a dramatic depletion of the Uba1a isoform in the *S100a8Cre*-CKO mutant NEs (*P* = 0.0001, Figure 1E). When using an alternative antibody sensitive to both Uba1a and Uba1b isoforms, we observed similar levels of Uba1a and Uba1b depletion (~31% of WT level, *P* = 0.012, Figure 1F). Although this antibody is sensitive to both isoforms in mouse BM cells, expression of the Uba1a isoform is detectable but weaker than that of the Uba1b isoform (Figure 1, F and G). Interestingly, in the mouse and human cell lines, Uba1a and Uba1b isoforms appeared to have similar expression levels (Figure 1G).

We also conducted quantitative reverse transcription PCR (qRT-PCR) assays and determined that residual levels of *Uba1* mRNA transcripts were approximately 50% of WT levels in total BM cells and approximately 30% of WT levels in purified NEs (Supplemental Figure 1B). Although there was slight leakage of *S100aCre* in purified monocytes, as determined by qRT-PCR (mRNA fold change: 0.78, *P* < 0.001; Supplemental Figure 1B), the protein level of residual Uba1 appeared grossly normal in the monocytes from *S100a8Cre*-CKO mutants (protein fold change: 0.87, *P* = 0.1979; Supplemental Figure 1C). Taken together, the results suggest that mouse Uba1 and human UBA1 were highly conserved and that our conditional disruption of the transcripts and protein isoforms by the Cre/fl approach was successful. Specifically, the residual levels of *Uba1* mRNA and Uba1 protein in NEs of *S100a8Cre*-CKO mutant mice were approximately 30% of the levels in WT mice.

### Conditional depletion of Uba1 in HSCs.

**Figure 2:**
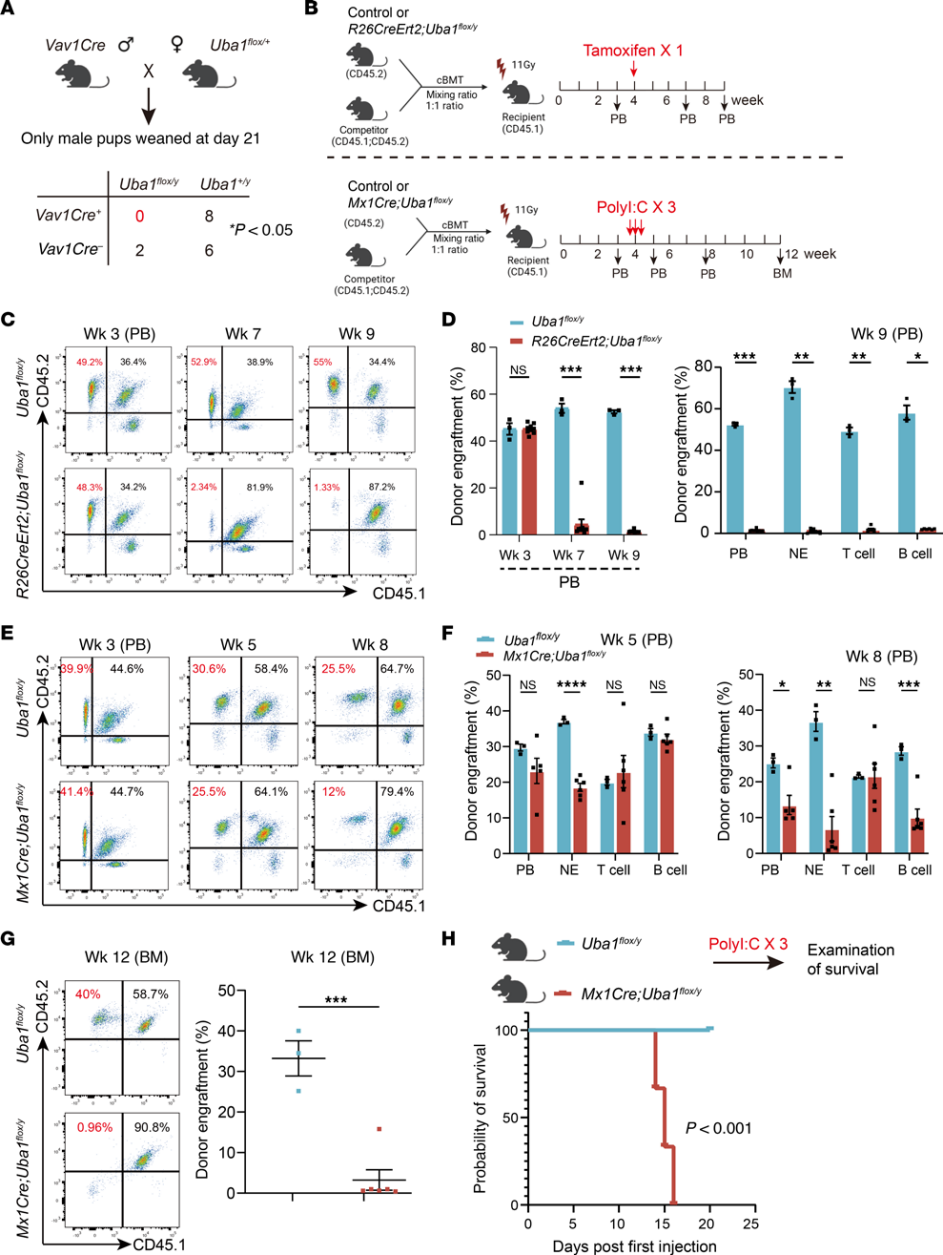
Conditional depletion of *Uba1* in HSCs. (**A**) Genetic crossing strategies to generate CKO mutants with depletion of *Uba1* in HSCs. *Vav1Cre*, *R26CreErt2*, and *Mx1Cre* were used for this goal. In the scheme, the strategy for the generation of *Vav1Cre Uba1^fl/y^* is shown as an example (upper panel). No *Vav1Cre Uba1^fl/y^* male pups were observed, suggesting lethality of *Vav1Cre*-mediated depletion of Uba1 in HSCs at the embryonic stages (lower panel; = *P < 0.05, χ^2^ test). (**B**) Generation of chimeric mice with depletion of Uba1 in 50% of the HSCs. The chimeric mice with BM reconstituted by cBMT assays through mixing *R26CreErt2 Uba1^fl/y^* or *Mx1Cre Uba1^fl/y^* donors (CD45.2^+^) with competitor donors (F1 mice: CD45.1^+^CD45.2^+^) as indicated (mixing ratio, 1:1). The recipient mice were CD45.1^+^. Induced depletion of *Uba1* in *R26CreErt2 Uba1^fl/y^* chimeric mice was conducted by feeding with tamoxifen (1 dose following cBMT). The induced depletion of *Uba1* in *Mx1Cre Uba1^fl/y^* chimeric mice was conducted by i.p. injection of PolyI:C (3 doses following cBMT). (**C** and **D**) Representative flow cytometric profiles and quantification of the donor engraftment in the *R26CreErt2 Uba1^fl/y^* chimeric mice at the indicated time points. *n* = 3~5 chimeric animals per group. (**E**–**G**) Representative flow cytometric profiles and quantification of the donor engraftment in the *Mx1Cre Uba1^fl/y^* chimeric mice at the indicated time points. **E** and **F** are results from flow cytometric analysis on PB cells, whereas **G** is the result from flow cytometric analysis on BM cells. *n* = 3~6 animals per group. (**H**) *Mx1Cre Uba1^fl/y^* primary mice proceeded to a quick death within 20 days after induction with PolyI:C (3 doses). Mice were 8~12 weeks old. *n* = 5 animals per group. Data represent the mean ± SEM. *P < 0.05, **P < 0.01, ***P < 0.001, and ****P < 0.0001, by Student’s *t*-test. *n* = 3~6 biological repeats.

As shown in Figure 2, A and B, we tried 3 different HSC targeting *Cre* lines to dissect the role of *Uba1* in HSCs, which are at the top the hematopoietic hierarchy, *Vav1Cre*, *Rosa26-Cre-Ert2* (labeled as R26CreErt2 here for simplicity), and *Mx1Cre*. *Vav1Cre* is active as early as E9 (36). After several rounds of breeding, we failed to obtain any male *Vav1Cre Uba1^fl/y^* pups (Figure 2A, n = 16 male pups in total, but *n* = 0 *Vav1Cre Uba1^fl/y^* male pups; χ^2^ test, *P* < 0.05). We then turned to generating *R26CreErt2 Uba1^fl/y^* or *Mx1Cre Uba1^fl/y^* mice by crossbreeding, as they remain alive when no drug is administered (tamoxifen for *R26CreErt2* and Poly I:C for *Mx1Cre*) (37, 38).

To dissect the role of *Uba1* in HSCs and following hematopoiesis, we constructed competitive BM transplantation (cBMT) assays as shown in Figure 2B. BM cells from donor mice *Uba1^fl/y^* were used as controls, along with BM cells from donor mice *R26CreErt2 Uba1^fl/y^* or *Mx1Cre Uba1^fl/y^* as competitors. Of note, upon tamoxifen induction, *R26CreErt2* was expressed in all cell types (including HSCs); upon polyI:C induction, *Mx1Cre* was specifically activated in HSCs and used for dissecting the roles of genes of interest in HSCs. As shown in Figure 2, C–G, activation of *R26CreErt2* by tamoxifen or activation of *Mx1Cre* by PolyI:C induced cell death in a large fraction of donor cells from *R26CreErt2 Uba1^fl/y^* or *Mx1Cre Uba1^fl/y^* BM, suggesting that *Uba1* was indispensable for HSCs and downstream of hematopoietic progenitor cells.

We were also interested in how long the primary *Mx1Cre Uba1^fl/y^* mice could survive after PolyI:C induction of *Uba1* depletion. As shown in Figure 2H, after administration of PolyI:C, the animals did not survive longer than 3 weeks (*n* = 5 mice per group, *P* < 0.001). Given that *Mx1Cre* is also active in multipotent progenitor cells (MPPs) (developmentally close to megakaryocyte-erythroid progenitors [MEPs]) and the halflife of RBCs is very short (~24 hours), one of the direct reasons for the death of *Mx1Cre Uba1^fl/y^* mutant mice was the short supply of RBCs. In conclusion, these results demonstrated that depletion of *Uba1* at the HSC level induced a quick and extensive death in almost all hematopoietic cell types.

### Conditional depletion of Uba1 in lymphoid cells or megakaryocytes.

**Figure 3:**
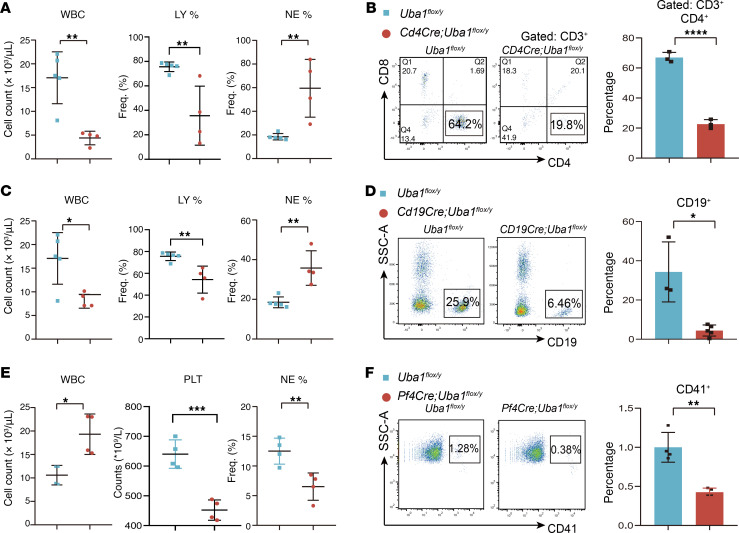
Conditional depletion of *Uba1* in CD4^+^ T cells, CD19^+^ B cells, and megakaryocytes. (**A**–**D**) Loss of *Uba1* in lymphoid cells (*Cd4Cre*- or *Cd19Cre*-induced) resulted in an obvious decrease in the number of leukocytes in PB. In **A** and **B**, the results are from *Cd4Cre Uba1^fl/y^* primary male mice; in **C** and **D**, the results are from *Cd19Cre Uba1^fl/y^* primary male mice. (**A** and **C**) Analysis of blood cells by the hematological analyzer. (**B** and **D**) Representative flow cytometric profiles and quantification of the flow cytometric results. LY, lymph cyte; PLT, platelet. (**E** and **F**) Loss of *Uba1* in megakaryocytes (*Pf4Cre*-induced) resulted in decreased numbers of platelets and megakaryocytes in PB. (**E**) Analysis of blood cells by the hematological analyzer. (**F**) Representative flow cytometric profiles and quantification of the flow cytometric results. Data represent the mean ± SEM. *P < 0.05, **P < 0.01, ***P < 0.001, and ****P < 0.0001, by Student’s *t*-test. *n* = 4~5 biological repeats. SSC-A, side scatter area; Freq., frequency.

We then tested the depletion of *Uba1* in mature blood cells in the hematopoietic and immune systems. Two *Cre* lines for lymphoid cells (*Cd4Cre* and *Cd19Cre*) (39, 40) and one *Cre* line for megakaryocytes (*Pf4Cre*) were used (41). As shown in Figure 3, A–D, a dramatically decreased amount of Cd4^+^ T cells or Cd19^+^ B cells was readily observed in *Cd4Cre Uba1^fl/y^* and *Cd19Cre Uba1^fl/y^* mice (cell depletion efficiency: 50%~70%). However, the mutant animals appeared normal and survived beyond 12 months in the specific pathogen–free (SPF) facility. As shown in Figure 3, E and F, approximately 50% depletion of megakaryocytes and platelets was also observed in *Pf4Cre Uba1^fl/y^* mice, but the mutant animals also appeared normal, and no VEXAS-like symptoms were observed in animals over 12 months of age. These results demonstrate that conditional depletion of *Uba1* in lymphoid cells or megakaryocytes successfully resulted in corresponding cell death but failed to induce VEXAS-like abnormalities in mice.

### Conditional depletion of Uba1 in monocytes by Lyz2Cre or Cx3cr1Cre.

**Figure 4:**
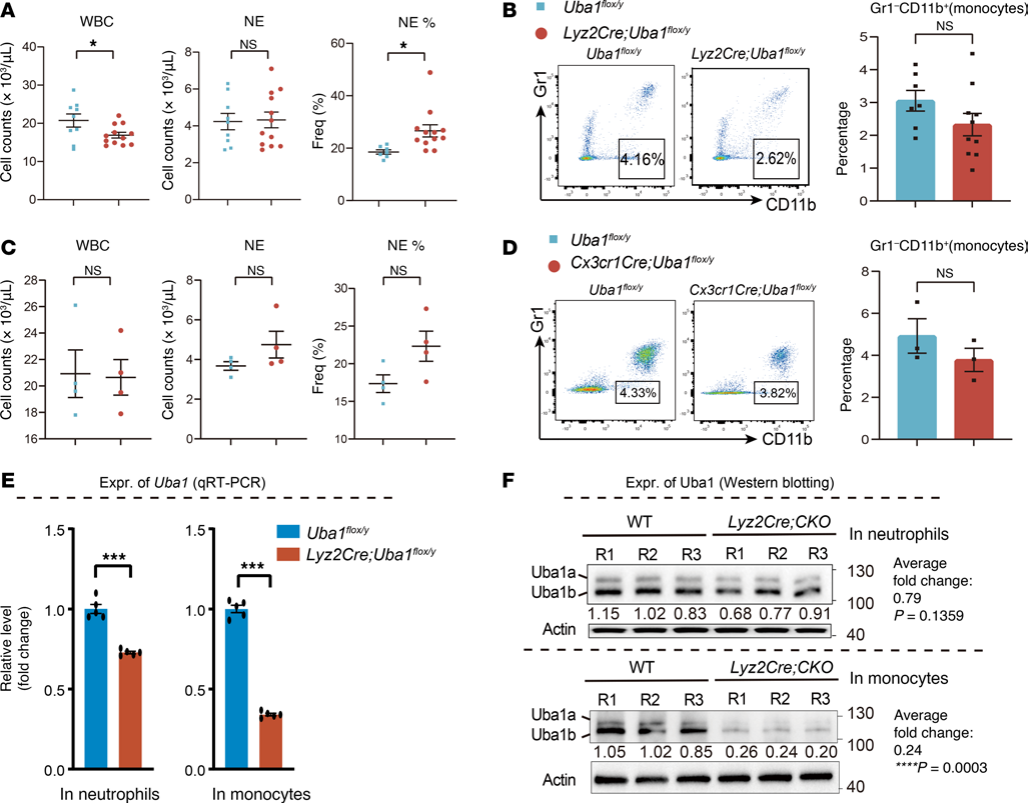
Conditional depletion of *Uba1* in monocytes and macrophages. (**A**–**D**) Mice with monocyte- and macrophage-specific loss of *Uba1* remained viable and did not develop VEXAS-like symptoms. In **A** and **B**, the results are from *Lyz2Cre Uba1^fl/y^* primary male mice; in **C** and **D**, the results are from *Cx3cr1Cre Uba1^fl/y^* primary male mice. (**A** and **C**) Analysis of blood cells by the hematological analyzer. (**B** and **D**) Representative flow cytometric profiles and quantification of the flow cytometric results. Note *Lyz2Cre Uba1^fl/y^*–CKO mutants manifested a subtly increased WBC count and an increased percentage of NEs. (**E**) Quantification of residual *Uba1* mRNA transcripts in NEs and monocytes from WT (*Uba1^fl/y^*) and *Lyz2Cre*-CKO (*Lyz2Cre Uba1^fl/y^*) mice by qRT-PCR. Compared with the controls, the residual average *Uba1* mRNA transcript levels in mutant NEs were approximately 75%, whereas the levels in mutant monocytes were 25% in *Lyz2Cre Uba1^fl/y^*–CKO mice, suggesting that *Lyz2Cre* was mainly active in monocytes and with subtle leakage in NEs. *n* = 5 biological repeats. (**F**) Quantification of residual Uba1 proteins in NEs and monocytes from WT (*Uba1^fl/y^*) and *Lyz2Cre*-CKO (*Lyz2Cre Uba1^fl/y^*) mice by Western blotting. Upper panel, expression of Uba1 in isolated NEs. Lower panel, expression of Uba1 in isolated monocytes. Note that in the *Lyz2Cre*-CKO mutants, a 76% reduction of Uba1 was detected in monocytes (average residual level: 24%, *P* = 0.0003), with only a 21% reduction of Uba1 in NEs (average residual level: 79%, *P* = 0.1359). Three independent biological repeats (R1, R2, and R3) were included. Data represent the mean ± SEM. *P < 0.05 and ***P < 0.001, by Student’s *t*-test. *n* = 3~11 biological repeats. Expr., expression.

We also tested depletion of *Uba1* in monocytes and macrophages, using 2 *Cre* lines for monocytes and macrophages: *Lyz2Cre* and *Cx3cr1Cre* (42, 43). As shown in Figure 4, A–D, there was no significant reduction of myeloid cells in *Lyz2Cre Uba1^fl/y^* or *Cx3cr1Cre Uba1^fl/y^* mice. Although we occasionally observed vacuoles in some myeloid cells from the blood of *Lyz2Cre Uba1^fl/y^* mice, both *Lyz2Cre Uba1^fl/y^* and *Cx3cr1Cre Uba1^fl/y^* mice appeared quite normal. To confirm the *Cre* activity induced by *Lyz2Cre*, we crossed *Lyz2Cre Uba1^fl/y^* with the *tomato-lox-GFP* line (referred to here as *lox-GFP* here for simplicity) (44). *Lyz2Cre lox-GFP* and *Lyz2Cre lox-GFP Uba1^fl/y^* mice were generated, respectively. Expression of GFP in *Lyz2Cre lox-GFP* and *Lyz2Cre lox-GFP Uba1^fl/y^* indicated *Lyz2Cre* activity and *Uba1* depletion in the *Lyz2Cre lox-GFP Uba1^fl/y^* compound mutants. As shown in Supplemental Figure 1, D and E, surprisingly, a loss of *Uba1* even resulted in a higher proportion of myeloid cells in the peripheral blood (PB).

To further characterize the myeloid cell depletion of *Uba1* by *Lyz2Cre*, we performed qRT-PCR and immunological blotting assays. As shown in Figure 4, E and F, a significant reduction of Uba1 was detected in the monocytes of *Lyz2Cre Uba1^fl/y^* mice (Uba1 residual expression was ~24% of control levels, *P* < 0.001). A slight reduction of Uba1 was also detected in NEs of *Lyz2Cre Uba1^fl/y^* mice compared with the controls (residual levels were ~75% by qRT-PCR, *P* < 0.001 and ~79% by Western blotting, *P* = 0.116; Figure 4, E and F), suggesting slight leakiness by *Lyz2Cre* in NEs in the *Lyz2Cre Uba1^fl/y^* mutants. Taken together, these results demonstrated that monocyte-based depletion of *Uba1* failed to induce VEXAS-like abnormalities in mice, and, intriguingly, somehow mature myeloid cells with loss of *Uba1* appeared to have an extended lifespan rather than vulnerability to cell death.

### Conditional depletion of Uba1 in NEs induces VEXAS-like phenotypes.

**Figure 5:**
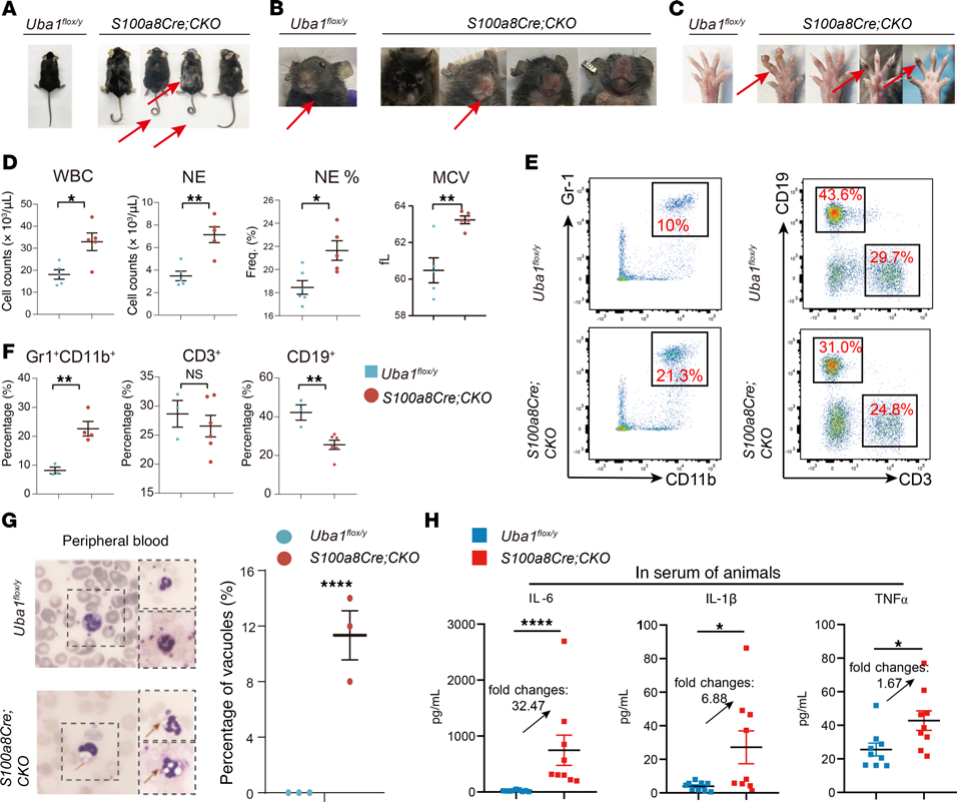
VEXAS-like autoinflammatory symptoms in *S100a8Cre*-CKO mice. (**A**) *S100a8Cre*-CKO mice manifested obvious dermatitis and hair loss on their back (penetration rate: 100%; *n* = 20). (**B**) *S100a8Cre*-CKO mice manifested flared noses (penetration rate: 100%; *n* = 20). (**C**) *S100a8Cre*-CKO mice manifested swollen paws (penetration rate: ~50%; *n* = 20), and the knuckles of their paws were pigmented (penetration rate: 100%; *n* = 20). In **A**–**C**, the penetration rate for checking the phenotypes was calculated at approximately 6–12 months of age. Stronger phenotypes and greater penetration rates were determined in aged mutant mice. (**D**) Aberrant hematological parameters in the PB of *S100a8Cre*-CKO mutants. (**E** and **F**) Representative flow cytometric profiles of PB and quantification of mature NEs (Gr1^+^CD11b^+^) and lymphoid cells (CD3^+^ or CD19^+^). (**G**) Approximately 10% of vacuolized NEs were identified in *S100a8Cre*-CKO mice. Giemsa staining and bright-light microscopy were performed on the blood samples. (**H**) Serum levels of proinflammatory cytokines IL-6, IL-1b, and TNF-α were upregulated in VEXAS-like mutant *S100a8Cre*-CKO mice. These cytokines were part of a 10-cytokine panel. See the full profiles in Supplemental Figure 7A. Data represent the mean ± SEM. *P < 0.05, **P < 0.01, and ****P < 0.0001, by Student’s *t*-test. *n* = 3~20 biological repeats.

As shown above, although the *Lyz2Cre* strain in the study demonstrated slight leakiness in NEs, it is not a strong and reliable *Cre* strain for targeting genes in NEs (left panel of Figure 4E; upper panel of Figure 4F). However, the fraction of NEs in BM (approximately 50%) was almost 5- to 10-fold that of monocytes (~5%~10%). We then tried depletion of *Uba1* in NEs using the *S100a8Cre* line, which is widely used for knocking out genes of interest in NEs (45). Most of the *S100a8Cre Uba1^fl/y^*–mutant mice appeared normal at 2~3 months of age, while a few of *S100a8Cre Uba1^fl/y^* mice appeared abnormal as early as 1 month of age (frequency: 10.6%, 5 of 47). When the *S100a8Cre*-CKO mice matured (~4 months old), we began to observe kinked tails (Figure 5A) and flared noses (Figure 5B), and such aberrations became more obvious in mice at older ages (i.e., 6~12 months of age). Additionally, hair loss on the backs of the mice, a sign of dermatitis, became apparent (Figure 5A). Furthermore, after examining the entire body, we also observed swollen toes and pigmentation on the knuckles of paws (including the front and the rear paws) in the *S100a8Cre*-CKO mutant mice (Figure 5C). The exact mechanism(s) by which the pigmentation appeared on the knuckles of the paws was not clear.

When we expanded the production of *S100a8Cre Uba1^fl/y^* male mutant mice by active breeding, we were also able to obtain female mutants (*S100a8Cre Uba1^fl/fl^*) among the offspring. The appearance of the bodies of the female mutant mice was identical to that described above for the male mutant mice, but the appearance of the bodies of all the different types of control animals was as normal as that of the WT animals, including the *S100a8Cre* strain itself (males and females), *Uba1^fl/y^* males, *Uba1^fl/+^* and *Uba1^fl/fl^* females, and *S100a8Cre Uba1^fl/+^* females. In addition, we alternatively husbanded the mutant mice (*S100a8Cre Uba1^fl/y^* or *S100a8Cre Uba1^fl/fl^*) in clean-grade air conditions for 12 months. The mutant mice in the clean-grade facility showed symptoms similar to those of the animals in the SPF facility, but no obvious symptoms of pneumonia or colitis were observed (*n* = 5). Taken together, these phenotypic observations confirmed that the kinked tails, flared noses, and knuckle pigmentation on paws were induced by *S100a8Cre*-mediated depletion of *Uba1* in the mutant male and female mice and that husbandry of the mutant mice in the SPF-grade or clean-grade facility resulted in body appearances similar to those described in Figure 5, A–C.

To examine any aberrant hematological parameters in the mutant mice with similarities to VEXAS syndrome in humans, we quantified the blood cells by hematological analyzers and flow cytometry. As shown in Figure 5D, the *S100a8Cre*-CKO mice had increased WBC counts (fold change: 1.5, *P* < 0.05), increased NE counts (fold change: 1.9, *P* < 0.01), and increased NE percentages (NE%) in WBCs (NE% fold change: 1.2, *P* < 0.05), which are hallmarks and indications of autoinflammatory diseases including VEXAS syndrome. Furthermore, a slight but significant increase in mean corpuscular volume (MCV) was also determined in *S100a8Cre*-CKO mutants (fold change: 1.05, *P* < 0.05) (Figure 5D). Of note, MCV is recognized as one of the key laboratory tests for VEXAS syndrome and an increase in MCV indicates anemia in the clinic (17). As shown in Supplemental Figure 2, A and B, we observed slightly impaired erythropoiesis in the BM of *S100a8Cre*-CKO mutants. The differences in RBC counts between the *S100a8Cre*-CKO mutants and the WT controls did not reach the bar of statistical significance. We also conducted flow cytometry to measure the changes in compartments of mature blood cells of *S100a8Cre*-CKO mutant mice or hematopoietic progenitor cells in the BM. As shown in Figure 5, E and F, the fraction of NEs (Gr1^+^CD11b^+^) in PB was increased, while that of B cells (CD19^+^) was decreased, consistent with results from the hematological analyzer (Figure 5D).

We also examined the existence of vacuoles in myeloid cells of the *S100a8Cre*-CKO mutants, one of the hallmarks of VEXAS syndrome (17). As shown in Figure 5G, we found that approximately 10% of myeloid cells (mainly NEs) had vacuolar characteristics. As shown in Supplemental Figure 2, C and D, we also observed rare occurrences of vacuolization in myeloid progenitor cells and erythroid cells of BM from the *S100a8Cre*-CKO mutants. Compared with the WT controls, the fraction of HSCs in the mutants was comparable, whereas that of granulocyte-macrophage progenitors (GMPs) and Lin^–^Sca1^–^cKit^+^ (LSK) cells appeared to be elevated (Supplemental Figure 3, A and B), suggesting inflammation regulation of hematopoiesis in the BM. Furthermore, H&E staining of skin, tail, and paw tissues indicated dermatitis and infiltration of inflammatory immune cells in the paws (Supplemental Figure 4, A–C). However, we did not observe obvious chondritis in the nose in *S100a8Cre*-CKO mice (relapsing polychondritis [RP] in the nose and ears is another hallmark of VEXAS syndrome in humans) (17). H&E staining of lung tissue identified minimal alteration of immune cells and stromal cells, but single-cell RNA-Seq (scRNA-Seq) analysis revealed increased inflammatory scores in the immune cells (Supplemental Figures 4 and 5). scRNA-Seq analysis of skin tissue, however, failed to identify obvious alterations, probably because of the insufficient capture of immune cells in the mutant skin tissues by our technical platforms or approaches (Supplemental Figure 6). In summary of the phenotypes, *S100a8Cre*-CKO mice had VEXAS-like symptoms, including inflammatory hematological parameters, vacuoles in NEs (~10%), dermatitis, and swollen paws. In addition, we frequently observed pigmentation on the knuckles of mutant mouse paws, which was not reported in the fingers or toes of patients with VEXAS syndrome. Finally, the mutant mice did not show visible RP in their ears, which was frequently reported in patients with VEXAS syndrome.

We then measured serological parameters in the mutant mice, as the level of proinflammatory cytokines is a key factor of several autoinflammatory diseases including VEXAS syndrome (17). We used a 10-cytokine panel to quantify proinflammatory and antiinflammatory cytokine levels. Among them, 3 proinflammatory cytokines — IL-1b, IL-6, and TNF-α — were significantly upregulated in serum, with fold changes between 2 and 40 (Figure 5H, n = 9 mice each for the WT group and the mutant group; see Supplemental Figure 7A for the full plots of the 10 cytokines in the tested mutant animals). Interestingly we observed a positive correlation between the 3 proinflammatory cytokines (IL-1b, IL-6, and TNF-α), but IFN-γ levels appeared unchanged (Supplemental Figure 7B), suggesting that expression of the 3 classical proinflammatory cytokines was systematically coordinated in the VEXAS-like mice. The results of the examination of mouse body appearance and analysis of blood cells and serum cytokine levels demonstrate that the *S100a8Cre*-CKO mutant mice manifested VEXAS-like autoinflammatory hemato-rheumatoid disorders.

### Ubiquitylation and cell survival advantage for mutant NEs.

**Figure 6:**
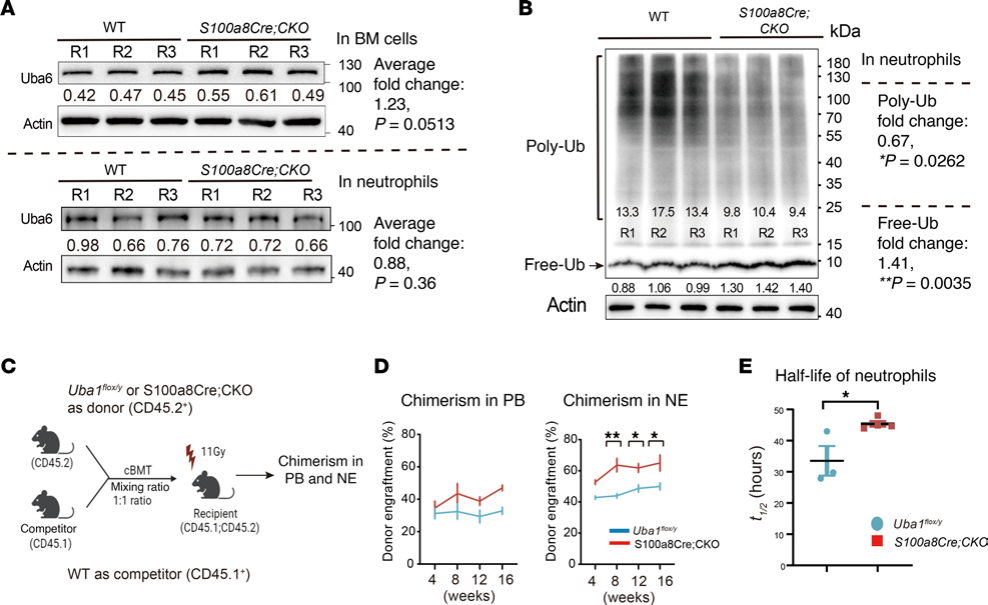
Ubiquitylation and cell survival in *S100a8Cre*-CKO NEs. (**A**) Expression of another E1 enzyme Uba6 appeared grossly normal in total BM cells (upper panel) and isolated NEs (lower panel) of *S100a8Cre*-CKO mice. R1/R2/R3 indicates 3 independent biological repeats. (**B**) Poly-Ub and free-Ub levels were quantified by Western blotting in isolated NEs from WT and *S100a8Cre*-CKO mice. Decreased poly-Ub and increased free-Ub levels were determined in *S100a8Cre*-CKO mice by the assays. R1/R2/R3 indicates three independent biological repeats. (**C** and **D**) Comparing the growth and survival advantage of WT NEs and mutant NEs by cBMT assays. BM cells from *S100a8Cre*-CKO and WT donors were isolated for performing the cBMT assays. **C**, the scheme for the cBMT experimental procedures. **D**, chimerism analysis in PB samples or in isolated NEs at the indicated time points post the cBMT. (**E**) Measuring the halflife (*t_1/2_*) of NEs in the PB by BrdU-pulse assays. See Methods for details about the full experimental procedures of BrdU staining, flow cytometry and computational analysis. Data represent the mean ± SEM. *P < 0.05, and **P < 0.01, by Student’s *t*-test. *n* = 3~5 biological repeats (**A**–**D**). *n* = 3~4 BrdU chasing assays for **E**, and each chasing assay used 6 biological repeats.

We also confirmed grossly normal expression of Uba6 in total BM cells and in purified NEs of *S100a8Cre*-CKO mice (Figure 6A). In addition, we demonstrated significantly downregulated poly-ubiquitylation (poly-Ub) levels and upregulated free ubiquitin (free-Ub) in mutant NEs from *S100a8Cre*-CKO mice (Figure 6B).

The increased NE counts in *S100a8Cre*-CKO mutants was intriguing, as this abnormality is in line with what we observed in *Lyz2Cre Uba1^fl/y^* mice. Flow cytometric profiling of hematopoiesis suggested a biased myelopoiesis (myeloid skewing) in *S100a8Cre*-CKO mice (Supplemental Figure 3, A and B). In addition, the body weights of *S100a8Cre*-CKO mutant mice were lower than those of WT control mice, while splenic weights were greater than those of WT controls, suggesting splenomegaly (a sign of leukemia or inflammation) in the mutant mice (Supplemental Figure 3C).

Although in NEs we observed slightly increased apoptotic activity, as determined by annexin V–marked flow cytometry (Supplemental Figure 3D), we functionally demonstrated an overall survival advantage of *S100a8Cre*-CKO myeloid cells, specifically for NEs, by 3 independent in vivo approaches. Similar to the *LyzCre lox-GFP* study (Supplemental Figure 1, D and E), we used the GFP reporter line and flow cytometric analysis in the first approach. The GFP reporter experiments suggest that a comparable or even higher fraction of Gr1^+^ cells or GFP^+^ cells in the *S100a8Cre*-CKO *lox-GFP* mice compared with that in the *S100a8Cre lox-GFP* controls (Supplemental Figure 3, E and F). For the second approach, we performed cBMT assays (see Figure 6C for the experimental scheme). As shown in Figure 6D, the donors from the *S100a8Cre*-CKO group manifested a phenotype of clonal hematopoiesis, which was also observed in patients with VEXAS syndrome (46). The cBMT assays suggest that the *S100a8Cre*-CKO NEs had a survival advantage. The third approach was to directly measure the halflife of NEs by BrdU staining and tracing (47). Compared with WT NEs, the *S100a8Cre*-CKO NEs had a significantly extended halflife (fold change: 1.35, *P* = 0.03; *t_1/2_* of mutant NEs = 45.3 ± 0.98 hours vs. *t_1/2_* of WT NEs = 33.52 ± 4.85 hours; *n* = 3~4 assay repeats per group, and 6 animals were recruited for a BrdU-chasing assay; Figure 6E and see Methods). Taken together, using 3 independent in vivo experimental assays, we demonstrated that mutant NEs lacking *Uba1* expression in *S100a8Cre*-CKO mice somehow had a growth or survival advantage in the BM over the WT controls.

### Disturbed NE homeostasis is revealed by assays on reactive oxygen species, proinflammatory cytokines, and phagocytosis.

**Figure 7:**
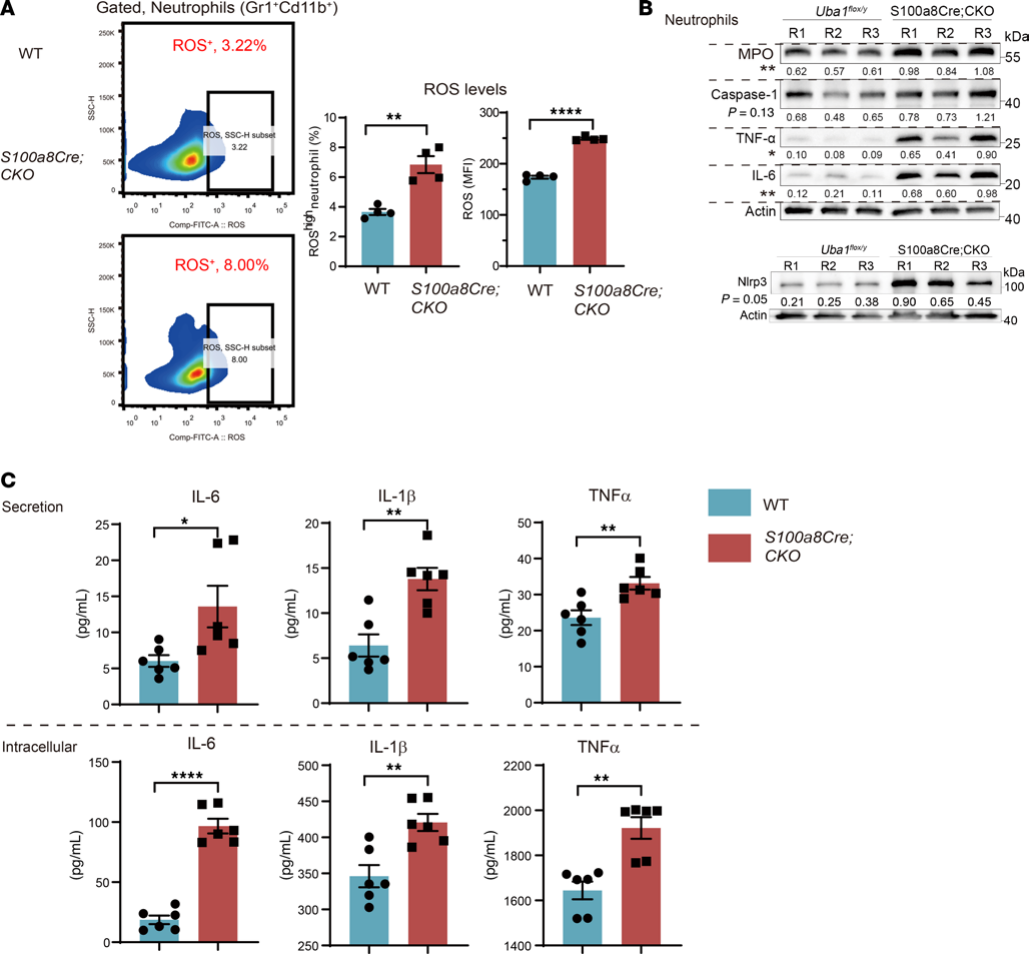
Cell-autonomous proinflammatory activation in *S100a8Cre*-CKO NEs. (**A**) Measurement of ROS production in NEs by flow cytometry. NEs from BM were gated by Gr1^+^Cd11b^+^ staining before the ROS analysis. Left panel shows representative flow cytometric profiles. Right panel shows quantitative results for ROS^hi^ NEs and ROS expression levels. *n* = 4 biological repeats per group. MFI, mean florescence of intensity. (**B**) Western blot analysis of inflammatory markers. MPO is a classic readout of inflammation; IL-6 and TNF-α are proinflammatory cytokines; Nlrp3 and caspase-1 are important components of the inflammasome complex or pathways. The fold changes of the tested proteins are between the ranges of 1.5 (caspase-1) and 7.25 (TNF-α). *P* values were labeled for each marker on the panel. R1/R2/R3 indicates 3 independent biological repeats. (**C**) Secretion and intracellular levels of the proinflammatory cytokines measured by ELISA. To measure the secretion level, 1 × 10^6^ purified NEs were cultured for 24 hours in a 96-well plate with 100 μL medium. To measure intracellular levels, 1 × 10^7^ purified NEs were incubated with RIPA lysis buffer. *n* = 4~6 biological repeats per group. Data represent the mean ±SEM. *P < 0.05, **P < 0.01, and ****P < 0.0001, by Student’s *t*-test. *n* = 3~6 biological repeats.

As *S100a8Cre* is mainly active in NEs, we wondered if any cell-autonomous abnormalities exist in *S100a8Cre*-CKO NEs with regard to reactive oxygen species (ROS) production, proinflammatory cytokine expression (i.e., IL-1b, IL-6, and TNF-α), inflammasome activation (i.e., NLRP3), NE extracellular trap (NET) formation, and phagocytosis capability on bacteria. Flow cytometry revealed that the mutant animals had increased fractions of ROS^hi^ NEs in the BM and increased expression of ROS in NEs (Figure 7A). Western blotting showed that mutant NEs had significantly increased expression of myeloperoxidase (MPO), IL-6, TNF-α, and Nlrp3 (fold change: 1.6–7.3; *P* ≤ 0.05). Expression of caspase-1 appeared to be increased (fold change: ~1.5; *P* = 0.13). We also performed ELISA to determine the cell-autonomous expression of the proinflammatory cytokines IL-6, IL-1b, and TNF-α in NEs. As shown in Figure 7C, both the secretion levels and intracellular levels of these 3 proinflammatory cytokines were significantly increased in mutant NEs, consistent with the serum results shown in the Figure 5H.

**Figure 8:**
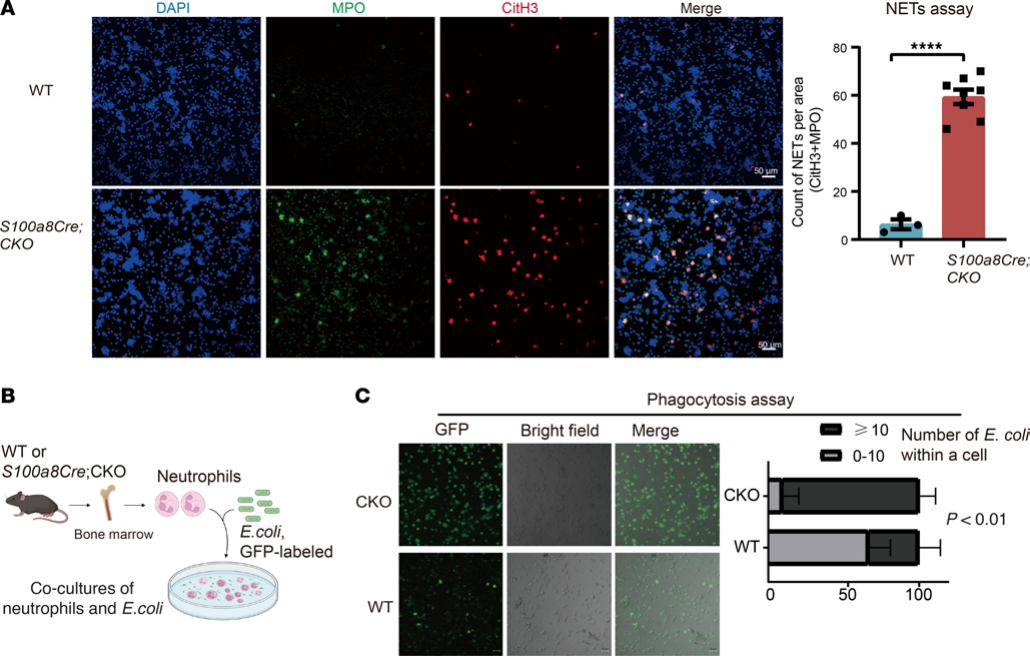
NETs formation and phagocytosis in *S100a8Cre*-CKO NEs. (**A**) Mutant NEs manifested increased level of NETs formation, which was determined by MPO and CitH3 staining. Purified NEs (1 × 10^6^) were cultured in a 24-well plate with 1,000 μL medium. Phorbol 12-myristate 13-acetate was added to the medium to promote cell attachment. After a 6-hour culture, cells were fixed and stained by MPO and CitH3 antibody. MPO and CitH3 double-positive cells were counted for a standard area. Left panel shows representative microscopy images; right panel shows the quantification results. *n* = 3 biological repeats for the WT group; *n* = 8 biological repeats for the *S100a8Cre*-CKO group. Scale bars: 50 μm. (**B** and **C**) Mutant NEs showed an increased phagocytotic capability when cocultured with bacteria *E. coli* (GFP^+^). (**B**) Scheme for the phagocytosis assay. Time-lapse photography was performed every 10 minutes after the mixing. GFP dots (indication of *E. coli*) within the NEs were counted 40 minutes after the mixing of NEs and bacteria. (**C**) Phagocytosis of *E. coli* was improved in *S100a8Cre*-CKO NEs compared with WT. Data are presented as the percentages of cells with certain counts of internalized bacteria (GFP^+^). *n* = 3 biological repeats per group. ****P < 0.000, by Student’s *t*-test. *n* = 3~8 biological repeats. Scale bars: 20 μm.

Aberrant activities of NETs and phagocytosis have been suggested in myeloid cells from patients with VEXAS syndrome (17). Staining with the NETs markers MPO and CitH3 revealed that the *S100a8Cre*-CKO NEs had increased counts of NETs in the ex vivo assays (Figure 8A). We experimentally examined the phagocytosis capability of the mutant NEs by coculturing NEs and GFP-labeled *E*. *coli* bacteria (see Figure 8B for the experimental scheme). As shown in Figure 8C, the mutant NEs appeared to have greater phagocytosis capabilities (*n* = 3 biological repeats, *P* < 0.01).

### Verification of NE-specific loss of Uba1 by single-cell transcriptomics analysis.

**Figure 9:**
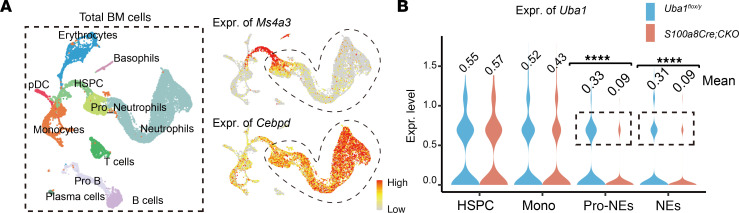
NE-specific depletion of *Uba1* in *S100a8Cre*-CKO BM revealed by scRNA-Seq analysis of BM cells. (**A**) BM cells from *Uba1^fl/y^* (WT) and *S100a8Cre*-CKO mice were subject for scRNA-Seq analysis. A total of 11 major hematopoietic cell types were annotated in the uniform manifold approximation and projection (UMAP) plot for dimension reduction. Of note, 2 types of NEs are highlighted: pro-NEs and NEs. As shown in the right panel, expression of *Ms4a3* (marker for GMP cells) and *Cebpd* (marker for primitive and mature NEs) marks the developmental continuity from HSPCs/GMPs to NEs. pDC, plasmacytoid dendritic cell. (**B**) Expression of *Uba1* in HSPCs, monocytes (Mono), pro-NEs, and NEs were quantified in *Uba1^fl/y^* (WT) and *S100a8Cre*-CKO scRNA-Seq datasets. Note that there is no reduction of *Uba1* expression in HSPCs from the *S100a8Cre*-CKO (residual level: 104%), a slight decrease of *Uba1* in monocytes (residual level: 82%) and a dramatic decrease of *Uba1* in pro-NEs or in NEs (residual levels: 27% and 29%, respectively). Each single cell of the indicated cell types was included for the Uba1 expression analysis. ****P < 0.0001, by Student’s *t*-test.

As shown in Figure 1, D–F, we experimentally determined NE depletion of Uba1 at both the mRNA and protein isoform levels (residual level: ~30%, indicating 70% depletion efficiency). To unbiasedly and orthogonally verify the exact cell type(s) with loss of *Uba1* expression in *S100a8Cre*-CKO, we took advantage of scRNA-Seq technologies. As shown in Figure 9A, a total of 11,204 and 10,054 BM cells were included in the uniform manifold approximation and projection (UMAP) plot comprising cells from 3 *Uba1^fl/y^* mice and 3 *S100a8Cre*-CKO mice. The major BM cell types were annotated (11 cell types in total; left panel of Figure 9A). The fractions of hematopoietic stem and progenitor cells (HSPCs) (which were mainly GMPs) and NEs were marked by expression of *Ms4a3* and *Cebpd*, respectively (right panel of Figure 9A). We detected a large reduction of *Uba1* transcripts in pro-NEs and NEs (residual levels: 27% and 29%, respectively, indicating ~70% depletion efficiency in both; Figure 9B) along with a subtle reduction of *Uba1* in monocytes (residual level: 83%, indicating ~20% depletion efficiency; Figure 9B). We did not detect a dramatic reduction of *Uba1* in other cell types including HSPCs (Figure 9B). These results provide orthogonal evidence suggesting a marked depletion of *Uba1* in NEs (pro-NEs and mature NEs) but not in other hematopoietic cell types (i.e., HSPCs and monocytes) in the *S100a8Cre*-induced CKO.

### Disturbed NE homeostasis is revealed by scRNA-Seq analysis.

**Figure 10:**
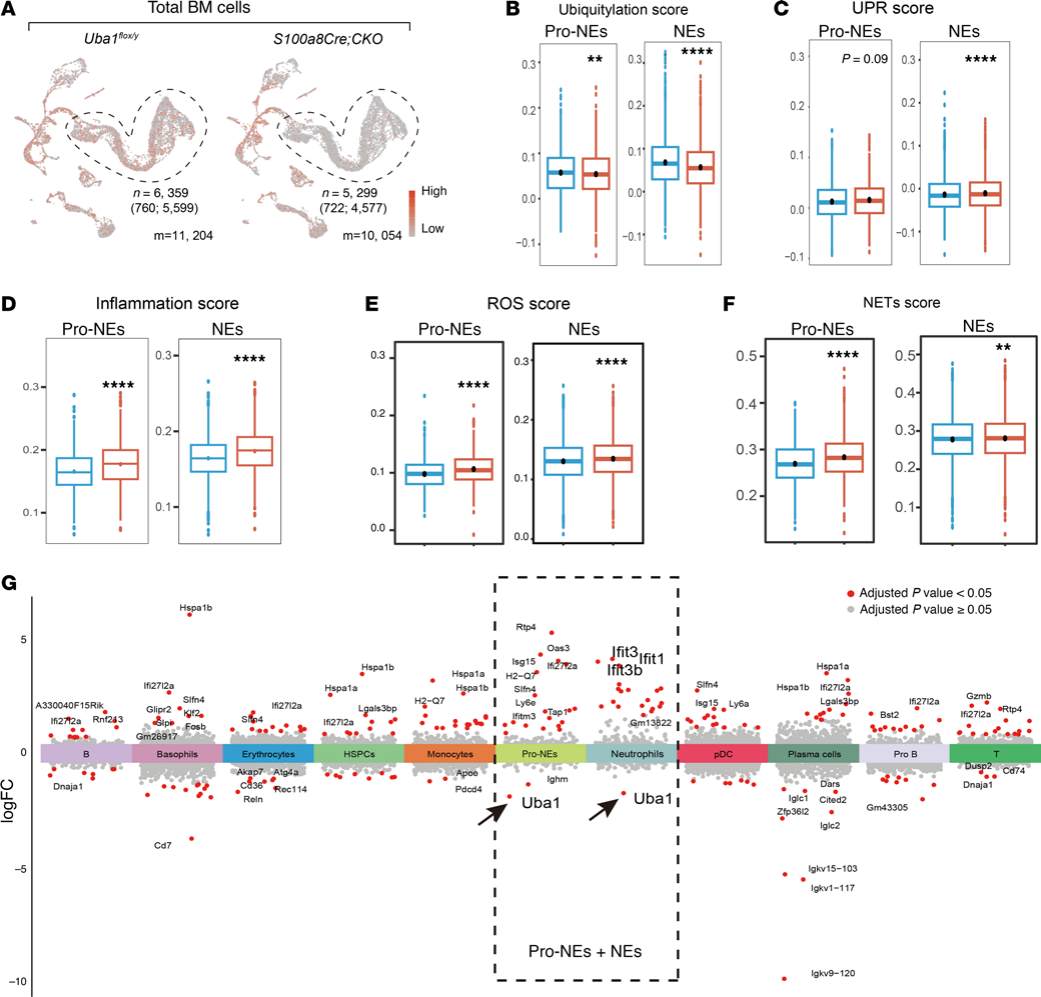
Disturbed NE homeostasis is revealed by single-cell computational analysis. (**A**) Expression of *Uba1* in WT and *S100a8*-CKO BM. Pro-NEs and NEs are highlighted in the UMAP plot and for downstream analysis of cell homeostasis. For WT, 760 pro-NEs and 5,599 NEs were included in the downstream analysis. For *S100a8Cre*-CKO, 722 pro-NEs and 4,577 NEs were included in the downstream analysis. (**B**) *S100a8Cre*-CKO mice had a lower ubiquitylation score in pro-NEs or NEs than did WT mice. The result was expected, as Uba1 is an E1 enzyme responsible for ubiquitin activation. (**C**–**F**) Loss of *Uba1* in NEs resulted in disturbed cellular homeostasis as indicated by an increased UPR score (**C**), inflammatory score (**D**), ROS score (**E**) and NETs score (**F**). (**G**) Manhattan plots of differentially expressed genes in each indicated cell type. As highlighted in the dotted line area, expression of *Uba1* was downregulated (arrows), and expression of certain proinflammatory genes (i.e., *Ifit1* and *Ifit3*) was upregulated in pro-NEs and NEs from *S100a8Cre*-CKO mice compared with those from WT control mice. Each single cell of the indicated cell types was included for the cellular activity analysis. **P < 0.01 and ****P < 0.0001, by Student’s *t*-test.

To further compare the BM activity in *Uba1^fl/y^* and *S100a8Cre*-CKO mice, we also took advantage of the scRNA-Seq datasets to measure cell homeostasis by scoring the activity of various functional aspects in NEs using the module score computational function in the Seurat toolkits (see Methods for details). The cells (pro-NEs and NEs) used for the scoring analysis are highlighted in Figure 10A. As shown in Figure 10B, the ubiquitylation scores were readily decreased in the pro-NEs and NEs of the mutants, consistent with the loss of function of Uba1 in the cells. Scoring the activity of the UPR, inflammation, apoptosis, necroptosis, ROS, and NETs formation further suggested disturbed cellular homeostasis (Figure 10, C–F, and Supplemental Figure 8, A–D). In Figure 10G, Manhattan plots highlight significant downregulation of *Uba1* and significant upregulation of the inflammation-related genes *Ifit1* and *Ifit3*. We also validated the results of scRNA-Seq analysis using the bulk RNA-Seq datasets on BM cells. As shown in Supplemental Figure 8, E and F, we detected upregulation of the inflammatory gene *Il1r1* and other genes in the inflammatory pathways.

In addition to the transcriptomics analysis, as shown in Supplemental Figure 9, A–H, liquid chromatography/mass spectrometry–based (LC/MS-based) proteomics analysis of BM cells from *Uba1^fl/y^* and *S100a8Cre Uba1^fl/y^* donors (*n* = 3 each) also demonstrated depletion of Uba1 in the BM (residual level: ~57.1%) and disturbed immune responses including a complementary response, an inflammatory response, and NETs formation. Taken together, results from scRNA-Seq datasets, bulk RNA-Seq datasets, and protein LC/MS datasets all indicated that depletion of *Uba1* in the *S100a8Cre*-CKO mutants resulted in various aspects of disturbed cellular or immunological activities including an increased UPR, ROS activity, and NETs formation. These immune-related abnormalities were also described in BM cells from patients with VEXAS syndrome (17).

### Inhibition of IL-1b/IL-1R1 signaling partially mitigates VEXAS-like symptoms.

**Figure 11:**
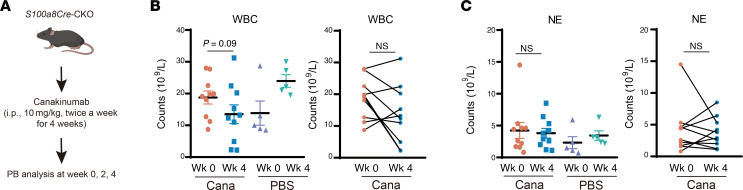
Treatment of VEXAS-like symptoms by inhibition of IL-1b and IL-1R1. (**A**) *S100a8Cre*-CKO mice were subjected to treatment with canakinumab (Cana) (anti–IL-1b). No pretests were performed. The canakinumab treatment regime for mice was as follows: 10 mg/kg, i.p., twice a week for 4 weeks. (**B** and **C**) At the drug treatment endpoint, we observed a reduction only of WBC counts, whereas NEs counts remained unchanged. MCV was also unchanged. *n* = 8 biological repeats per group. See also Supplemental Figure 8 for the treatment with anakinra (anti–IL-1R1), which also only partially mitigated the VEXAS-like symptoms in *S100a8Cre*-CKO mice.

It is well known that autoinflammatory diseases are generally modulated by IL-1 signaling (48). We tried treatment for the *S100a8Cre*-CKO mutants with anakinra (a peptide-like antagonist for IL-1R1) or canakinumab (a monoclonal antibody for IL-1b). As shown in Figure 11, A–C, and Supplemental Figure 10, A–C, the present treatment regime with anakinra or canakinumab only partially mitigated the symptoms in *S100a8Cre*-CKO: WBC counts were decreased, but NE counts appeared unchanged.

### Genetic loss of Morrbid ameliorates VEXAS-like symptoms.

**Figure 12:**
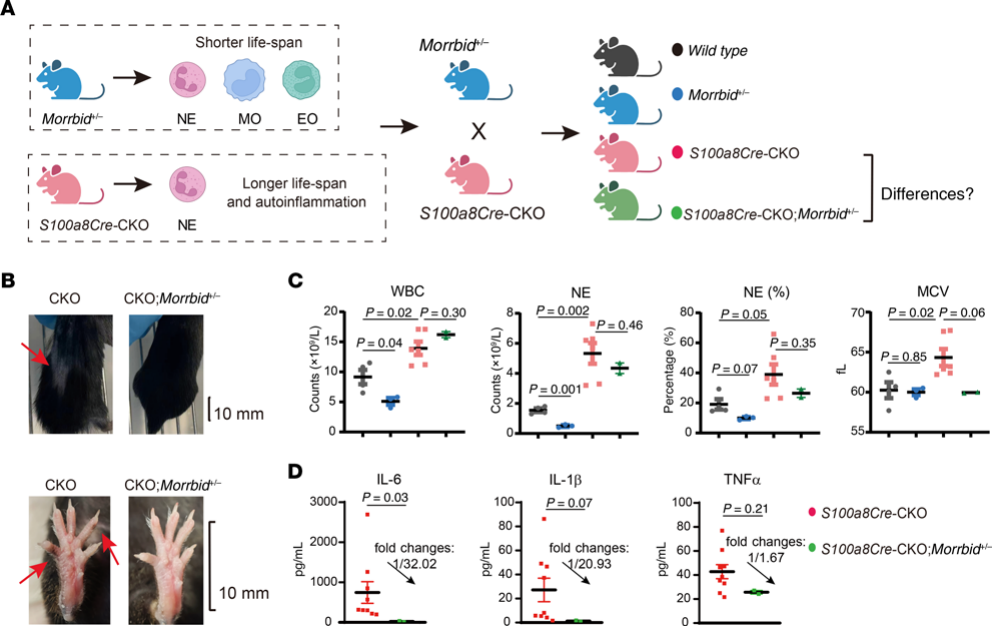
Treatment of the VEXAS-like symptoms by genetic loss of *Morrbid*. (**A**) *S100a8Cre*-CKO mice were bred with *Morrbid^+/–^* mice to test the role of *Morrbid* in the autoinflammatory disease VEXAS syndrome. The human-mouse conserved long noncoding RNA (lncRNA) gene *Morrbid* is a prosurvival regulator for myeloid cells, especially for NEs, monocytes (MO), and eosinophils (EO). Similar to the homozygous *Morrbid^–/–^* mouse, the heterozygous mutant *Morrbid^+/–^* has a similarly shorter lifespan of myeloid cells. We generated the compound mutants *S100a8Cre*-CKO *Morrbid^+/–^* mice along with other control mice to determine the role of *Morrbid* in VEXAS syndrome. (**B**) Hair loss on the back and pigmentation on the paw knuckles were mitigated in the compound mutants *S100a8Cre*-CKO *Morrbid^+/–^* mice. Scale bars: 10 mm. (**C**) Hematological analysis of PB cells. MCV, NE counts, and NE percentages all showed a decreased trend, indicating the mitigation of VEXAS-like symptoms by genetic loss of *Morrbid*. (**D**) Serum levels of the proinflammatory cytokines IL-6, IL-1b, and TNF-α were downregulated in the compound-mutant *S100a8Cre*-CKO *Morrbid^+/–^* mice. Fold changes and *P* values are labeled in **C** and **D**. Data represent the mean ± SEM. *P* values were determined by Student’s *t*-test. *n* = 2~9 biological repeats per group.

In our previous anti-leukemia studies, we demonstrated that the human-mouse conserved long noncoding RNA *Morrbid* is a prosurvival regulator for myeloid cells and has a role in myeloid-lineage leukemogenesis (49–52). In addition, elevated expression of *Morrbid* is readily detected in the transcriptomics dataset of BM cells in the *S100a8Cre*-CKO mutants (Supplemental Figure 10D). According to the rationale shown in Figure 12A, we assessed the role of *Morrbid* by generating the compound mutant *S100a8Cre*-CKO *Morrbid^+/–^* (*Morrbid^+/–^* heterozygous and *Morrbid^–/–^* homozygous mice had similarly short myeloid cell lifespans). As shown in Figure 12, B–D, genetic loss of *Morrbid* also partially mitigated VEXAS-like symptoms including decreased WBC counts and MCV, disappearance of pigmentation in the paw knuckles, and decreased serum levels of proinflammatory cytokines.

## Discussion

**Figure 13:**
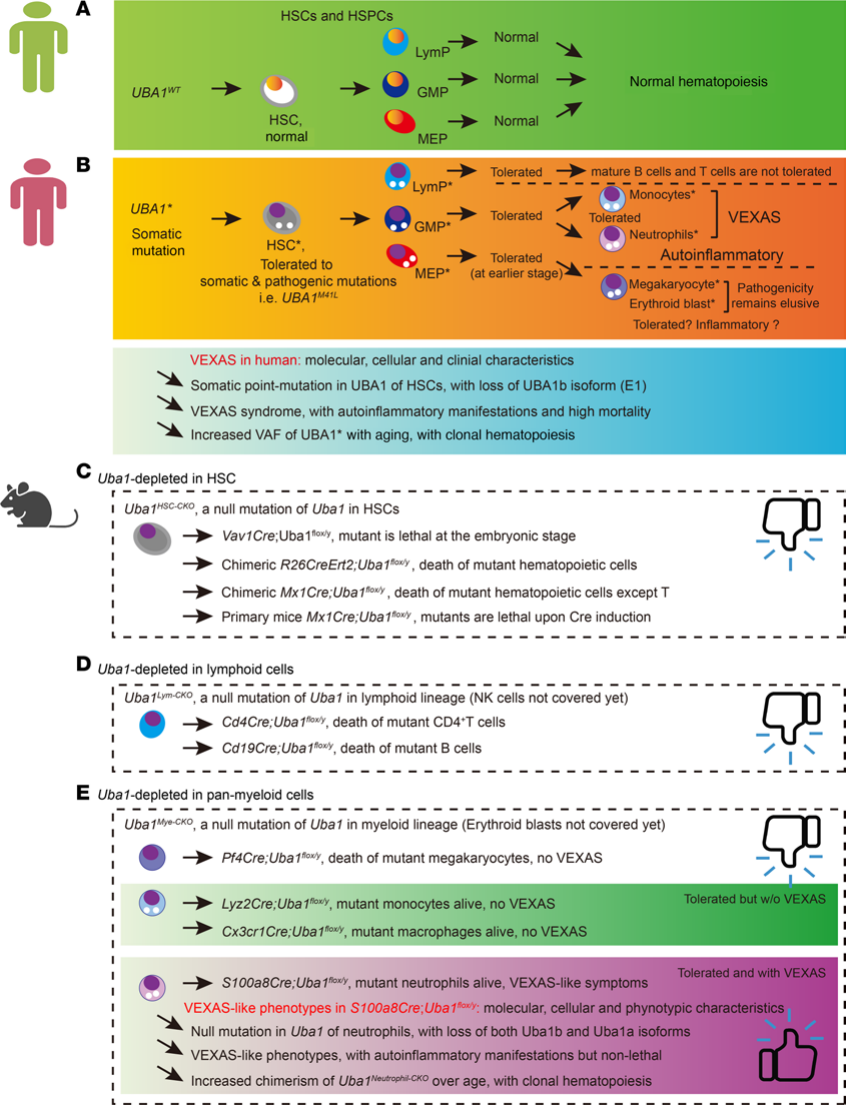
Summary of the study and a comparison with VEXAS-related clinical studies. (**A** and **B**) Normal hematopoiesis (**A**) and VEXAS-conditioned hematopoiesis (**B**). According to the published clinical results, as summarized in the lower panel of **B**, patients with VEXAS manifest 3 typical features. (i) Somatic and pathogenic *UBA1* point mutation in HSCs (typically at the M41 site, indicating a loss of the cytoplasm isoform UBA1b). The VEXAS-like outcomes are likely attributable to the combination effect of all blood cells. (ii) Autoinflammatory symptoms at older ages (around ≥40 years of age for older males). (iii) Increased VAF of mutant *UBA1* and occurrence of clonal hematopoiesis. (**C**–**E**) The outcomes of 9 different CKO lines used in the study are summarized in **C**, **D**, and **E**. Except for monocytes and NEs, we demonstrate that most of the hematopoietic progenitor and mature cells were not tolerant of the null-version mutation of *Uba1*. Importantly, we demonstrate that monocytes and NEs survived with an approximately 30% residual level of Uba1. However, monocyte depletion of Uba1 in *Lyz2Cre*-CKO and *Cx3cr1Cre*-CKO mice did not lead to VEXAS-like symptom development like in NE depletion of Uba1 in *S100a8Cre*-CKO mice. As summarized in the lower panel of **E**, VEXAS-like symptoms in *S100a8Cre* CKO mice also included 3 typical features. (i) A null version of *Uba1* mutation in NEs, indicating that both nucleic and cytoplasm isoforms were deficient and that the effect was mainly attributable to the mutant NEs. (ii) Autoinflammatory symptoms were typically apparent at 4 months of age and extended to older ages (i.e., ≥6 months of age), but the lifespan of the *S100a8Cre*-CKO mutant animals was grossly normal. (iii) The cBMT experiments showed increased fraction of mutant NEs and the occurrence of clonal hematopoiesis. See also the Discussion in the main text and Supplemental Tables 1–4 for further comparisons through different angles.

As illustrated in Figure 13, we aligned the findings from the clinical studies of VEXAS syndrome in human (Figure 13, A and B) and the results of our study in mice (Figure 13, C–E). Supplemental Tables 1–4 provide 4 angles covering the comparison of various aspects of human VEXAS syndrome and our mouse modeling in detail. Overall, it is persuasive that VEXAS syndrome in humans is induced by the comprehensive and synthetic consequence of somatic, pathogenic, and loss-of-function of *UBA1* mutations in HSCs; however, the 9 CKO murine models in the present study recapitulated the effects of *Uba1* loss (null mutation) in certain hematopoietic cell types. Thus, our study shows that the big difference between appearance of VEXAS in humans and the phenotypes of various CKO and chimeric mice could be summarized as a holistic and synthetic effect in humans versus a limited and cell-type–specific effect in mice.

Interestingly it was suggested by some clinical studies that mutant monocytes with somatic point mutations in *UBA1* may contribute to VEXAS autoinflammatory symptoms in humans (17, 53, 54). However, our study does not support the argument that mutant monocytes alone with a null mutation of *Uba1* intrinsically play a role in VEXAS-like syndrome in mice. Among the nine *Uba1*-CKO mutants described here, only *S100a8Cre*-CKO mice had induced VEXAS-like symptoms. In addition to inducing gene KO in mature monocytes and macrophages*, Lyz2Cre* is reported to have leakage in NEs to a certain extent. As *Lyz2Cre*-CKO mice don’t show a phenotype as strong as that of *S100a8Cre*-CKO mice, Cre activity in NEs for *Lyz2Cre* is probably not as strong as that for *S100a8Cre*. An alternative explanation is that the timing of *S100a8Cre* is probably earlier than that of *Lyz2Cre* for NE depletion of Uba1. Using qRT-PCR and immunological blotting, we experimentally verified that *Lyz2Cre* does have slight leakage in NEs, but the residual Uba1 protein levels were maintained at as high as approximately 78%, while residual of Uba1 protein levels in monocytes were approximately 24% (Figure 4, E and F). Lack of abnormalities in *Cx3cr1Cre*-CKO mice provided further evidence that monocyte or macrophage loss of *Uba1* was not sufficient to induce the VEXAS-like phenotypes in murine models. In addition, the fraction of monocytes and macrophages in the BM and PB was much less than that of NEs, which may represent another reason for the lack of a VEXAS-like phenotype in *Lyz2Cre*-CKO and *Cx3cr1Cre*-CKO mice. Through scRNA-Seq measurement of gene expression and cell activity, we again validated the NE-specific loss of *Uba1* in *S100a8Cre*-CKO mice and disturbed cellular and immunological activities in various aspects (ubiquitylation, UPR, inflammation, ROS and NETs), which are related to the intrinsic function of Uba1 and NEs (Figures 9 and 10).

We are aware that *S100a8Cre*-CKO mice (NE-only–based *Uba1^null^*) do indeed lack the same the point mutations as in humans (chimeric but all types of hematopoietic cell–based *UBA1^mut^*). However, both mutations are loss-of-function ones, which is one of the key genetic etiologies of VEXAS syndrome. Another key genetic etiology is the threshold of the variant allele value of *UBA1^mut^*, which merits future study in the lab as well as in the clinic. Reversing such loss-of-function defects could guide future development of interventions for this important biological process mediated by UBA1. By disrupting the function of Uba1 in nearly all of the major cell types in the BM, our study showed that null mutations of *Uba1* in NEs rather than in other tested hematopoietic cells are critical for inducing VEXAS-like symptoms. Nonetheless, installing a point mutation in mouse HSCs by gene editing is undoubtedly necessary to fully recapitulate VEXAS symptoms in mice, which was suggested in a recent meeting abstract and in a mouse myeloid cell line (34, 35).

Human UBA1 and mouse Uba1 are highly conserved and critical for eukaryotic cells. A quick and extensive death of hematopoietic cells took place in the HSC-wise depletion of *Uba1* (*Vav1Cre*, *R26CreErt2* and *Mx1Cre*-mediated, respectively), suggesting HSCs with a null mutation of Uba1 (HSC-*Uba1^null^*) are not tolerated at all. In the context of our HSC-targeting *Uba1^null^* studies, tolerance of somatic mutations in human HSCs (i.e., *UBA1^M41L^*) is quite intriguing and indicates that further functional studies are required to determine whether a compensation function of the UBA1a isoform or other compensation mechanisms (i.e., UBA6) exist in human HSCs with *UBA1^M41L^*. Of note, 2 such types of compensation mechanisms are unlikely to occur in *Uba1^null^* mice, since the Uba1a isoform was also depleted and Uba6 expression appeared normal (Figure 1, D and E, and Figure 6A). Importantly, no VEXAS-like phenotypes were observed in the 2 HSC-targeting chimeric mice with various chimeric fractions as analyzed in cBMT assays using *Mx1Cre*-CKO or *R26CreErt2*-CKO donor cells. These results further suggest that HSC-based *UBA1^M41L^* was unequal to HSC-based *UBA1^null^*. Tolerated or not tolerated and pathogenic or not pathogenic represent 2 key future questions to ask for individual *UBA1* variant among the full spectrum of mutations identified in the available VEXAS syndrome cohorts (16, 18, 23, 26, 28).

Although we provided data on the intolerance of megakaryocytes by *Pf4Cre-*CKO, the cell-type–dependent tolerance and pathogenicity of erythroid blasts and megakaryoblasts among other intermediate myeloid and lymphoid progenitors and blast cells, will still be one of the interesting questions to ask in the future (i.e., experimental and functional examination). For this goal, the use of VEXAS-conditioned hematopoietic cells for a direct test under the ex vivo or in vitro experimental settings is also necessary. In addition, inducible *Cre* lines targeting hematopoietic progenitors (i.e., GMPs, MEPs, and lymphoid progenitors), rather than HSCs, will also be worthwhile to dissect the cell-type–specific role of Uba1 along the full trajectory and each branch and hierarchy level of hematopoiesis.

In contrast to intolerance of *Uba1^null^* for HSCs, we uniformly observed the tolerance of *Uba1^null^* for monocytes and NEs, which was also observed in human mutant monocytes and NEs with *UBA1^M41L^* (17). Although the flow cytometry–based apoptosis profile of mutant NEs suggests greater apoptotic activity, we provide alternative in vivo evidence supporting the survival advantage of mutant NEs when comparing them with normal NEs: (a) hematological cell counts in the PB, (b) cBMT assays, (c) halflife tracing by BrdU staining, and (d) lox GFP–based genetic tracing. We speculate that increased expression of the proinflammatory cytokines IL-6, IL-1b, and TNF-α is the driver of the survival advantage of mutant NEs and that a positive feedback loop occurs in VEXAS disease progression. In our previous studies, such a positive feedback loop model has been proposed to explain how *TET2* mutations induce clonal hematopoiesis where downstream regulators including IL-1b, IL-6, *Morrbid*, and *Ptx3* are important for the *TET2* mutation–mediated hematopoietic and immunological abnormalitie (49–51, 55). Of note, the “clonal hematopoiesis” observed in the cBMT assay in the study was simply attributed to NE loss of *Uba1* in the donor cells. It would be interesting to clinically test in the future if mitigating clonal hematopoiesis and such positive feedback loops confer any benefits for patients with VEXAS syndrome (29, 46, 55).

Since VEXAS syndrome in humans is a synthetic and holistic effect of all aspects of mutant hematopoietic cells carrying somatic *UBA1* mutations, in the future, it will therefore be required to generate compound *Cre* lines to test whether a stronger phenotype manifests (i.e., generating *Lyz2Cre S100a8Cre* *Uba1^fl/y^*). Furthermore, anakinra, an inhibitor of IL-1/IL-1R1 signaling, and ruxolitinib, an inhibitor of JAK2 signaling, have been reported to treat VEXAS syndrome (56, 57). It will be interesting to test if the combination of these 2 drugs alleviates the autoinflammatory symptom in *S100a8Cre*-CKO mice. In addition, in our recent work on *Pstpip2*-KO induced autoinflammatory disease, chronic multifocal osteomyelitis, and *Ncf2*-KO–induced chronic granulomatous disease, we demonstrated that genetic loss of *Morrbid* mitigated these 2 autoinflammatory diseases too (58, 59). In addition to its function in anti-leukemia, targeting *Morrbid* (i.e., anti-*Morrbid* by antisense oligos) may represent a valuable strategy for inhibiting autoinflammation in clinical management. In the future, additional preclinical studies that comprehensively target IL-6, IL-1b, and TNF-α will be necessary to assess the efficacy of antiinflammation treatment for VEXAS.

### Limitation of the study.

In the present study, we introduced a null version of mutation in *Uba1* rather than a point mutation like *Uba1^M41L^* in murine models. Furthermore, multiple *Uba1^non-M41^* variants were reported. However, it should be cautioned that the pathogenicity of those non-*M41* variants requires functional experiments to validate these findings (16, 18, 23, 26, 28). Our animal model studies provide an indirect but complementary and timely understanding of VEXAS syndrome. However, the present study did not cover the respective roles of *Uba1* for erythroid cells, natural killer cells, mast cells, eosinophils, or basophils. To fully dissect the role of *Uba1* in the hematopoietic system, additional *Cre* lines will be required to breed with the *Uba1* fl strain. Furthermore, our preliminary phagocytosis experiments indicated that the mutant NEs appeared to have stronger NETs and phagocytotic capabilities. Given the easy maintenance of the *S100a8Cre Uba1^fl/y^* colonies, it will be interesting to test whether the mutant mice manifest any aberrations in additional inflammatory or traumatic challenges including pathogen-host immunity, trauma, and cancer immunity (50, 58, 60).

### Conclusion.

In conclusion, we report on the VEXAS-like mouse model *S100a8Cre Uba1^fl/y^* for mimicking an autoinflammatory disease recently identified in human, VEXAS syndrome. Using the other 8 CKO models, we also demonstrated that targeting certain hematopoietic cell types, rather than NEs, failed to manifest VEXAS-like symptoms. These results clearly document the intrinsic and diversified functions of Uba1 in mammal hematopoiesis and immunity-related cells and indicate the cell-type–dependent pathogenicity of *UBA1^mut^* in human VEXAS syndrome. The VEXAS-like mouse model *S100a8Cre Uba1^fl/y^* will provide a further understanding of VEXAS syndrome and warrants future development of effective treatments for the autoinflammatory disease highly prevalent among older men.

### Footnote.

When our study was under revision, Molteni et al. published their meeting abstract as a peer-reviewed report (61). Interestingly, their experimental assays on gene-edited HSCs suggest that in cell culture, erythroid cells are not tolerated of the *UBA1^M41T^* (p.Met41Thr) point mutation.

## Methods

Additional details on the mouse strains and experimental procedures are provided in the Supplemental Methods.

### Statement regarding sex as a biological variable.

As VEXAS syndrome is a disorder mostly affecting older men, we mainly generated male CKO mutant mice (*Cre Uba1^fl/y^*) and collected the tissue samples from the male mutant mice for the assays described in the study. As we stated in the Results, for *S100a8Cre*-mediated CKO models, the male mutant *S100a8Cre Uba1^fl/y^* and the female mutant *S100a8Cre Uba1^fl/fl^* mice manifest identically aberrant body appearances, but we still mainly used *S100a8Cre Uba1^fl/y^* mice for all analyses described in this work and used *Uba1^fl/y^* mice as WT controls.

### Study approval.

The animal study was approved by Tianjin Medical University Laboratory Animal Resource Center, and experiments were conducted according to the protocol.

### Data availability.

The Supporting Data Values file for the Figures is provided in the Supplemental materials. Raw bulk RNA-Seq and scRNA-Seq data in this study have been deposited in the China National Center for Bioinformation/Beijing Institute of Genomics database at https://ngdc.cncb.ac.cn/bioproject/browse/PRJCA025038 (GSA: CRA015841). Proteomics data and scRNA-Seq Matrix data have been deposited in the OMIX database at https://ngdc.cncb.ac.cn/omix/release/OMIX006177 (OMIX006177) and https://ngdc.cncb.ac.cn/omix/release/OMIX006178 (OMIX006178).

### Statistics.

Statistical analysis was performed using GraphPad Prism 9 (GraphPad Software). If not stated otherwise, data in the figures are presented as the mean ± SEM. One-way ANOVA with an uncorrected Fisher’s test or 2-tailed Student’s *t* test was used to determine significant differences between groups. A *P* value of less than 0.05 was considered statistically significant.

## Author contributions

ZC is the guarantor of the study. ZC conceived, designed, and supervised the study, analyzed the data, and wrote the manuscript. GD generated mouse models, monitored phenotypes, validated the NE or monocyte depletion of Uba1 and phagocytosis assays, and validated normal expression of Uba6. JL generated mouse models, monitored phenotypes, validated the NE or monocyte depletion of Uba1, the poly-ubiquitin, free ubiquitin, cytokine, and ROS levels, and inflammation pathways. WJ, HZ, YW, and ZW generated mouse models, monitored phenotypes, performed scRNA-Seq and proteomics analysis, assisted with the protein and RNA experiments, and analyzed data. KX, JZ, LM, YM, LCC, QZ, HW, WW, and YF assisted with the experiments and maintenance of the mouse models. All authors edited and approved the manuscript.

## Funding support

- Tianjin Medical University Talent Program, to ZC.
- State Key Laboratory of Experimental Hematology, to ZC.
- Tianjin Key Laboratory of Inflammatory Biology, to ZC.
- National Natural Science Foundation of China (NSFC) (82371789 and 82170173), to ZC.

## Supplementary Material

Supplemental data

Unedited blot and gel images

Supplemental tables 1-4

Supporting data values

## Version 1 09/04/2025

In-Press Preview

## Version 2 11/03/2025

Electronic publication
