# Supplementary material for: UBA1-depleted neutrophils disrupt immune homeostasis and induce VEXAS-like autoinflammatory disease in mice
Source: J Clin Invest. 2025 Sep 4;135(21):e193011. doi: 10.1172/JCI193011 (PMC12578396; doi:10.1172/JCI193011)
Supplement: Supplemental data [file jci-135-193011-s032.pdf]

SUPPLEMENTARY MATERIALS

TITLE

UBA1-depleted neutrophils disrupt immune homeostasis and induce VEXAS-like autoinflammatory disease in mice

Authors

Ge Dong[1,2,3,#], Jingjing Liu[1,2,3,#], Wenyan Jin[1,2,3, Δ], Hongxi Zhou[1,2,3, Δ], Yuchen Wen[1,2,3, Δ], Zhiqin Wang[1,2,3, Δ], Keyao Xia[1,2,3], Jianlin Zhang[1,2,4], Linxiang Ma[1,2,3], Yunxi Ma[1,2,4], Lorie Chen Cai[3], Qiufan Zhou[5], Huaquan Wang[5], Wei Wei[6], Ying Fu[7], Zhigang Cai[1,2,3,4,5,6,*]

This Supp. Material file contains:

1. **HIGHLIGHTS of the Study**
2. **METHOS**
3. **LEGENDS for Figure S1-S10**

HIGHLIGHTS of the Study

• WHAT IS ALREADY KNOWN ON THIS TOPIC:

VEXAS syndrome is a recently identified hematological and immunological disease prevalent in adult man but rarely in adult woman. Somatic mutations in the E1-enzyme encoding gene *UBA1* in hematopoietic stem cells is the driver on the top of the genetic etiology of the disease. However, the major pathogenic cell type(s) responsible for VEXAS syndrome has not been experimentally examined. Furthermore, genetically reliable mouse models recapitulating the disease are lacking.

• WHAT THIS STUDY ADDS:

i. Using nine different conditional-knockout (CKO) murine models, we interrogated the pleiotropic phenotypes caused by loss of the ubiquitin activation enzyme E1/Uba1 in different hematopoietic cell types;

ii. Our results demonstrated that among the nine tested CKO mutants, only neutrophil loss of *Uba1* results in VEXAS-like autoinflammatory disease;

iii. The VEXAS-like symptoms in the *S100a8Cre-CKO* mutant mice include: in the mutant animals, (a) increased counts of white blood cells and neutrophils, (b) increased percentage of neutrophils, (c) increased serum level of proinflammatory cytokines (IL-1β, IL-6 and TNFα); and in the mutant neutrophils, (d) observation of vacuoles, (e) increased survival, (f) increased expression of MPO, ROS and NETs, (g) increased cell-autonomous expression and secretion of proinflammatory cytokines (IL-1β, IL-6 and TNFα), and (h) increased phagocytosis;

iv. Pharmacological treatments with IL-1 inflammatory pathway inhibitors Anakinra or Canakinumab or genetic loss of the myeloid pro-survival regulator *Morrbid* partially mitigated the VEXAS-like symptoms in the *S100a8Cre-CKO* mutants.

• HOW THIS STUDY MIGHT AFFECT RESEARCH, PRACTICE OR POLICY

The study reports a technical strategy of developing the murine models for VEXAS syndrome. In addition, the study dissects cellular and molecular mechanisms, especially the cell-type-dependent tolerance and pathogenicity of loss of function of *Uba1*, for the occurrence of the autoinflammation diseases in mice. The study provides translational implications in etiology and potential treatment choices for the newly-identified haemato-rheumatoid syndrome in clinical management.

METHODS

Mice

As the VEXAS syndrome is a disorder mostly affecting aged men, we mainly generated male CKO mutant mice (*Cre; Uba1*$^{flox/y}$) and collected the tissue samples from the male mutant mice for the following studies described below.

The *Uba1*$^{flox/y}$ mice were generated by the CRISPR/Cas9 approach. The Cre mice were described previously and purchased from Jax Laboratory. The dTomato-lox-GFP mice (named here as lox-GFP for simplicity) were from Jax Laboratory. The Cre and flox strained used in the study all are in C57/B6 background. Except *Vav1Cre*-CKO, we used other CKO male mice at the age of 8 to 24 weeks and the *Uba1*$^{flox/y}$ or corresponding *Cre*-positive males in the same litter or with same ages as controls. Animal experimentation was performed in accordance with protocols approved by the Animal Care and Use Committee of Tianjin Medical University.

Cell lines

The human HEK293-T cell line, K562, and mouse Raw264.7 cell line were purchased from Chinese Academy of Medical Sciences (Shanghai, China). The HEK293-T and Raw264.7 cell were cultured with Dulbecco's modified Eagle's medium (DMEM) (MeilunBio, MA0212) containing 10% fetal bovine serum (FBS) (ExCell Bio, FSD500). The K562 cell was cultured with RPMI-1640 medium (MeilunBio, MA0215) containing 10% FBS.

H&E Staining

The tissue samples were fixed in 4% paraformaldehyde. After the dehydration in ethanol, the tissue was embedded in paraffin then sectioned with a thickness of 4 μm. The histopathological feature was tested via H&E staining.

Flow cytometry

The bone marrow (BM) cells and peripheral blood mononuclear cells (PBMCs) were flushed out with FACS buffer. Single-cell suspensions were treated with red blood cell lysis buffer, stained, and analyzed using FACS Canto II (BD Biosciences). For mature cell analysis, staining with antibodies against CD19 (#152410, BioLegend), CD3 (#100206, BioLegend), Gr-1 (#108408, BioLegend), CD11b (#101206, BioLegend), CD4 (#100510, BioLegend), CD8a (#100712, BioLegend), CD41 (#133904, BioLegend). For early hematopoietic cell analysis, cells were incubated with biotinylated antibodies against the lineage (Lin) markers: CD11b (#101206, BioLegend), Ter119 (#116208, BioLegend), Gr-1 (#108408, BioLegend), CD3 (#100206, BioLegend), B220 (#103208, BioLegend), and the fluorescence-conjugated antibodies: c-Kit (#105812, BioLegend), Sca-1 (#108126, BioLegend), CD34 (#11-0341-85, BioLegend), CD150 (#115912, BioLegend), CD48 (#103424, BioLegend), and CD16/32 (#101318, BioLegend).

Competitive bone marrow transplantation (cBMT) and chimerism assays

Recipient animals (BoyJ, CD45.1) were lethally irradiated (7Gy plus 4Gy) one day prior to transplantation (intravenous tail injection) of donor cells. For generating chimeric mice mimicking hematopoietic clonal expansion, *Cre;Uba1*$^{flox/y}$ donor cells and F1 donor cells were mixed at a ratio of 1: 1 (100K:100K). 4 weeks after BM *R26CreErt2;Uba1*$^{flox/y}$ transplantation animals were continuously administered by gavage with tamoxifen at a dose of 15mg/ml in 200ul of corn oil for 5 days.

Mx1Cre;Uba1$^{flox/y}$ was injected with PolyI:C at a dose of 1.5mg/ml in 100ul of PBS by intraperitoneal injection three times on alternate days.

Neutrophil phagocytosis assays

Bone marrow cells were isolated from age and sex matched mice (12-week old), followed by neutrophil enrichment subsequently using the density gradient centrifugation (Sigma-Aldrich Cat. No.11191, No.10771) according to the manufacturer's instructions. The enriched neutrophil sample purity was assessed by flow cytometry with CD11b and Ly6G, confirming expected purity. GFP-labeled *E. coli* bacteria were grown overnight to an OD600 of 0.5, and subsequently co-cultured with neutrophils. For co-culture, 5x105 cells in 0.5mL of phenol red-free RPMI (Gibco) supplemented with 10% pooled mouse serum were plated per 35mm glass bottom imaging dish (Mattek corporation) and mixed with GFP-labeled *E. coli* bacteria at the ratio 1:10. Time lapse imaging was performed using the confocal scanning microscope (Leica) and six fields of view were captured over 40 min per sample. Phagocytosis was analyzed in FIJI software after stitching the six fields of view together.

Immunofluorescence Staining of Neutrophil Extracellular Traps (NETs)

Neutrophils were isolated from mouse bone marrow using density gradient centrifugation. The cells were resuspended in RPMI 1640 medium, and 1 mL of neutrophil suspension (at a density of approximately 1×10^6 cells/mL) was seeded into 24-well plates containing sterile glass coverslips. The cells were stimulated with 100 ng/mL PMA (phorbol 12-myristate 13-acetate) and incubated for 6 hours at 37°C in a 5% CO_2 incubator. After stimulation, cells were fixed with 4% paraformaldehyde at room temperature for 30 minutes, followed by permeabilization with 0.2% Triton X-100 for 10 minutes at room temperature. Non-specific binding was blocked using 10% goat serum for 60 minutes at 37°C. Primary antibodies anti-CitH3 (Abcam, #281584) and anti-MPO (R&D, #AF3667) (40 ul each) were added and incubated overnight at 4°C. After washing with PBS, cells were incubated with appropriate fluorescent secondary antibodies for 2 hours at room temperature in the dark. Nuclei were stained with DAPI for 5 minutes in the dark at room temperature, followed by mounting with anti-fade mounting medium.

Enzyme linked immunosorbent assay (ELISA)

ELISA detection for ten inflammatory factors (IFN-γ, IL-1β, IL-2, IL-4, IL-5, IL-6, KC/GRO, IL-10, IL-12p70, TNF-α) were performed using Proinflammatory Panel-1 (mouse) Kits (MSD, NJ, USA). Mouse blood samples were collected using an empty tube without anticoagulant, left to stand at 4°C for 2 hours (to avoid shaking and prevent hemolysis), centrifuged for 20 minutes (4°C, 3000g), and collected the supernatant. The samples were frozen at -80°C. Neutrophils were isolated from bone marrow using density gradient centrifugation. One portion of the cells was lysed in RIPA buffer supplemented with 1% PMSF to extract cytoplasmic proteins, while the other portion was seeded into 96-well plates and cultured in 1640 medium for 24 hours to collect the culture supernatant. Before the experiment, the reagent was moved to room temperature for 30min. According to the kit instructions, we added 25-50uL of standard, quality control, and sample sequentially at room temperature (700rpm, 1h). Then we added 25-50uL of detection antibody and incubate at room temperature (700rpm, 1h). The samples underwent 3 washes with 150uL cleaning solution, followed by the addition of

150uL of plate reading solution before machine analysis. Analysis was carried out using MSD Discovery Workbench (Version 4.0).

BrdU pulse-phase assay for measuring half-life of neutrophils

Mice were injected intraperitoneally with 2 mg of BrdU (Sigma-Aldrich) at the indicated time points (Day -8 to Day -1, one dose each day prior to the BrdU flow cytometry assays). To detect BrdU incorporation, cells from the blood were stained with neutrophil surface makers CD11b (#101206, BioLegend) and Ly6G (#127608, BioLegend). Cells were then fixed, permeabilized, and finally stained intracellularly with APC-conjugated anti-BrdU antibody according to the manufacturer's protocol before analysis with flow cytometry.

Drug Treatments for the mutants

Anakinra (recombinant interleukin-1 receptor IL-1R1 antagonist): the *S100a8Cre-CKO* mice were treated by i.p. injection with Anakinra (37mg/kg) or PBS for four times a week. Blood examination was recorded at Week 0, 2, and 4 post the treatment. Canakinumab (recombinant IL-1β monoclonal antibody): the S100a8Cre-CKO mice were treated by i.p. injection with Canakinumab (10mg/kg) or PBS for twice a week. Blood examination was recorded at Week 0, 2, and 4. At Week 4 post the treatment, flow cytometry was used to detect neutrophils in the blood.

Total protein extraction

The samples were taken out in the frozen state and put on ice. The samples were suspended in protein lysis buffer (8M urea,1% SDS) which included appropriate protease inhibitor to inhibit protease activity and the mixture were treated by high-flux tissue grinding machine for 3 times, 40 s each. Then the mixture was incubated on ice for 30 min, during which was vortex mixed for 5-10 s every 5 min. After centrifugation at 16000g at 4°C for 30 min, the concentration of protein from the supernatant collected were determined by Bicinchoninic acid (BCA) method by BCA Protein Assay Kit (Thermo Scientific). Protein quantification was performed according to the kit protocol.

Western blot analysis

Proteins were harvested from BM cells with RIPA Lysis Buffer (Beyotime, Jiangsu, China) supplemented with phenylmethyl sulfonyl fluoride (PMSF) protease inhibitor and phosphatase inhibitor. Total protein concentration was determined by BCA Protein Assay Kit (Beyotime, Jiangsu, China), denatured protein samples of appropriate quality of proteins were subjected to sodium dodecyl sulfate polyacrylamide gel electrophoresis (SDS-PAGE) and then transferred to PVDF membranes. Then membranes were later blocked with 5% skimmed milk, and incubated were immunodetected with specific antibodies against UBA1a/b (#4891; Cell signaling technology, USA)， antibodies against Uba1a (#4890; Cell signaling technology, USA), antibodies against UBA6 (#13386; Cell signaling technology, USA), antibodies against Free Ubiquitin and Polyubiquitin (#43124; Cell signaling technology, USA), antibodies against Ubiquitin (#3936; Cell signaling technology, USA), antibodies against MPO (#ab208670, Abcam), Caspase-1 (#24232; Cell signaling technology, USA), antibodies against TNF-α (#11948; Cell signaling technology, USA), antibodies against IL-6 (#12912; Cell signaling technology, USA), antibodies against NLRP3 (#15101; Cell signaling technology, USA), Actin (AC026, ABclonal, China) overnight at 4 °C. Protein bands were visualized by the MINICHEMI Imaging System (Surwit, Hangzhou,

China) and the ECL Substrate (GenStar, Beijing, China).

Bulk RNA-Seq Data Analysis

RNA-seq analysis pipeline was conducted in house and has been described in our previous studies (55). Volcano plots were used to show the fold changes and log-adjusted p-values for DEGs. We used the R package "clusterProfiler" to perform Gene Ontology (GO) analyses. "enrichGO" and genome-wide annotation packages "org.Mm.eg.db" were needed. A p-value < 0.05 was considered significant enrichment.

Single-cell RNA-sequencing (scRNA-seq) analysis

--data acquiring and processing

Single-cell RNA-Seq libraries were prepared using SeekOne® MM Single Cell 3' library preparation kit (No. K00104, SeekGene). Briefly, the appropriate number of cells were loaded into the flow channel of SeekOne® MM chip which had 170,000 microwells and allowed to settle in microwells by gravity. After removing the unsettled cells, sufficient Cell Barcoded Magnetic Beads (CBBs) were pipetted into flow channel and also allowed to settle in microwells with the help of a magnetic field. Next excess CBBs were rinsed out and cells in MM chip were lysed to release RNA which was captured by the CBB in the same microwell. Then all CBBs were collected and reverse transcription were performed at 37°C for 30 minutes to label cDNA with cell barcode on the beads. Further Exonuclease I treatment were performed to remove unused primer on CBBs. Subsequently, barcoded cDNA on the CBBs was hybridized with random primer which had reads 2 SeqPrimer sequence on the 5' end and could extend to form the second strand DNA with cell barcode on the 3' end. The resulting second strand DNA were denatured off the CBBs, purified and amplified in PCR reaction. The amplified cDNA product was then cleaned to remove unwanted fragments and added to full length sequencing adapter and sample index by indexed PCR. The indexed sequencing libraries were cleanup with SPRI beads, quantified by quantitative PCR (KK4824, KAPA Biosystems) and then sequenced on illumina NovaSeq 6000 with PE150 read length. The raw gene expression matrices of the scRNA-seq data were read and combined, converting them into a Seurat object using the Seurat package (version 4.4.0) in R software (version 4.2.0). During the quality control process, we retained cells meeting the following criteria: nFeature_RNA >200 and nFeature_RNA < 5000; and UMIs derived from the mitochondrial genome < 5. We excluded genes related to mitochondria from the datasets. We performed the function FindVariableFeatures (Seurat) to detect the features with highest coefficient of variation (CV). By default, we chose the top 2000 variable features to calculate a PCA matrix with 30 components and transported the PCA matrix into Harmony (version 1.2.0) to integrate single-cell data gene expression matrix and correct the batch effect.

--unsupervised clustering and annotation of cell types in the UMAP plots

The Harmony matrix would be used for unsupervised clustering by building the nearest neighbor graph and Louvain algorithm. The unsupervised clustering (resolution = 0.3) for identifying the main cell types, including the HSPCs (Ms4a3, Cebpd), Pro Neutrophils (Camp, Chil3), Neutrophils (Mmp8), Monocytes (F13a1, Ms4a6c), Erythrocytes (Hba-a2, Hbb-bt), pDC (Siglech, Cox6a2), Basophils (Prss34, Mcpt8), B (Cd19), Pro B (Vpreb3), T (Cd3e), Plasma cell (Jchain, Iglc2).

--construction of gene signatures and scoring bioactivities in each cell

We integrated classic gene sets to characterize different bioprocesses, generating gene

lists for subsequent analyses. The function 'AddMouduleScore' in the Seurat package was employed to calculate the average expression levels for each cluster. All signatures were binned based on the average expression.

Proteomic analysis

--protein reductive alkylation and digestion

Take protein samples 100 μg and add TEAB (Triethylammonium bicarbonate buffer), which the final concentration of TEAB is 100 mM. Then add TCEP (tris (2-carboxyethyl) phosphine) to the final concentration of 10 mM and react for 60 min at 37 °C. Following add IAM (Iodoacetamide) to the final concentration of 40 mM and react for 40 min at room temperature under dark conditions. Add a certain percentage (acetone: sample v/v = 6:1) of pre-cooled acetone to each sample and to settle for 4 h at -20 °C. After centrifugal for 20 min at 10000 g, the sediment was collected and add 100 μL 100mM TEAB solution to dissolve. Finally, the mixture was digested with Trypsin overnight at 37 °C added at 1:50 trypsin-to-protein mass ratio.

--protein extraction and quantification

Samples were thawed on ice and processed using a high-throughput tissue grinder in a lysis buffer containing 8M urea and 1% SDS to ensure thorough lysis. Additionally, to prevent proteolytic degradation during the process, a cocktail of protease inhibitors was added. Samples were homogenized in three cycles of 40 seconds each, followed by a 30-minute incubation on ice with vertexing every 5 minutes to enhance protein extraction. The mixture was then centrifuged at 16,000g for 30 minutes at 4°C to separate the soluble proteins from cellular debris. The supernatant was carefully collected, and protein concentration was determined using the Bicinchoninic Acid (BCA) Protein Assay Kit (Thermo Scientific), following the manufacturer's instructions to ensure accurate quantification.

--protein digestion

Proteins were diluted to a final concentration of 100 μg in 100 mM Triethylammonium bicarbonate (TEAB) and reduced with 10 mM Tris(2-carboxyethyl) phosphine (TCEP) at 37°C for one hour. Subsequent alkylation was performed with 40 mM iodoacetamide (IAM) in the dark at room temperature for 40 minutes to protect sensitive amino acid residues. Proteins were then precipitated using cold acetone at a ratio of 6:1 (v/v), incubated at -20°C for 4 hours to enhance precipitation, and centrifuged. The resulting pellet was resuspended in 100 mM TEAB for enzymatic digestion. Trypsin was added at a 1:50 enzyme-to-protein ratio and the mixture was incubated overnight at 37°C to achieve complete digestion.

--peptide processing and desalting

Following digestion, peptides were vacuum dried and resolubilized in 0.1% trifluoroacetic acid (TFA) for desalting. This step was critical to remove salts and other contaminants that could interfere with subsequent mass spectrometric analysis. Desalting was performed using HLB cartridges, and peptides were eluted, collected, and again subjected to vacuum drying. Peptide quantification was performed using a specialized Peptide Quantification Kit from Thermo Fisher Scientific, following the provided protocol to ensure consistent results.

--phospho-peptide and ubiquitylated peptide enrichment (UPE)

For selective enrichment of phospho-peptides, the peptide was first dissolved using Binding/Wash Buffer, adding a Binding/Wash Buffer equilibrated column, centrifugation, and then the redissolved peptide was added to the column and incubated for 30min. After incubation, add Binding/Wash Buffer cleaning column, centrifuge; add LC_MS grade water to clean the column, then add Elution Buffer stripping twice; finally, add equal volume of 20% TFA acidification, stage-tip desalination, and drain by vacuum concentrator. For ubiquitylated peptides, enrichment was carried out using Anti-K-ε-GG antibody beads provided by Cell Signal Technology, which specifically recognize diglycine-modified lysine residues. Both types of peptides were bound to their respective beads under optimized conditions, washed to remove non-specifically bound peptides, and the bound peptides were eluted. The eluted peptides were then desalted using C18 Stage Tips to prepare them for LC-MS/MS analysis.

--LC-MS/MS analysis

Peptide mixtures were analyzed using a state-of-the-art VanquishNeo system coupled to an Orbitrap Astral mass spectrometer (Thermo, USA). Separation was achieved on a C18-reversed phase column with a gradient of acetonitrile in 0.1% formic acid. Peptides were analyzed in data-independent acquisition (DIA) mode, which allows for a comprehensive survey of all peptides present, with a mass range set from m/z 100 to 1700.

--data analysis

Raw data files were processed using the Spectronaut software (Version 18), with a stringent false discovery rate (FDR) of ≤0.01 for protein and peptide identifications to ensure high confidence in the results. Quantitative analysis was based on the integrated peak areas of at least six peptides per protein and three daughter ions per peptide, providing robust quantification metrics.

--bioinformatics analysis

Differentially expressed or modified proteins were identified using the Majorbio Cloud platform, with statistical significance set at $p < 0.05$ and fold change thresholds. Functional annotations and pathway analyses were conducted using the Gene Ontology (GO) and Kyoto Encyclopedia of Genes and Genomes (KEGG) databases to elucidate biological implications of the proteomic changes observed.

Statistical analysis

Statistical analysis was conducted by using GraphPad 9. Comparisons between 2 groups were determined by using a two-tail *Student's* t-test or by using *Wilcoxon* test. Comparison of multiple groups were determined by using an ANOVA analysis of variance with the Dunnett multiple comparisons test. Results with $p < 0.05$ were considered statistically significant.

LEGENDS for Supplemental Figures: Figure S1 to Figure S10

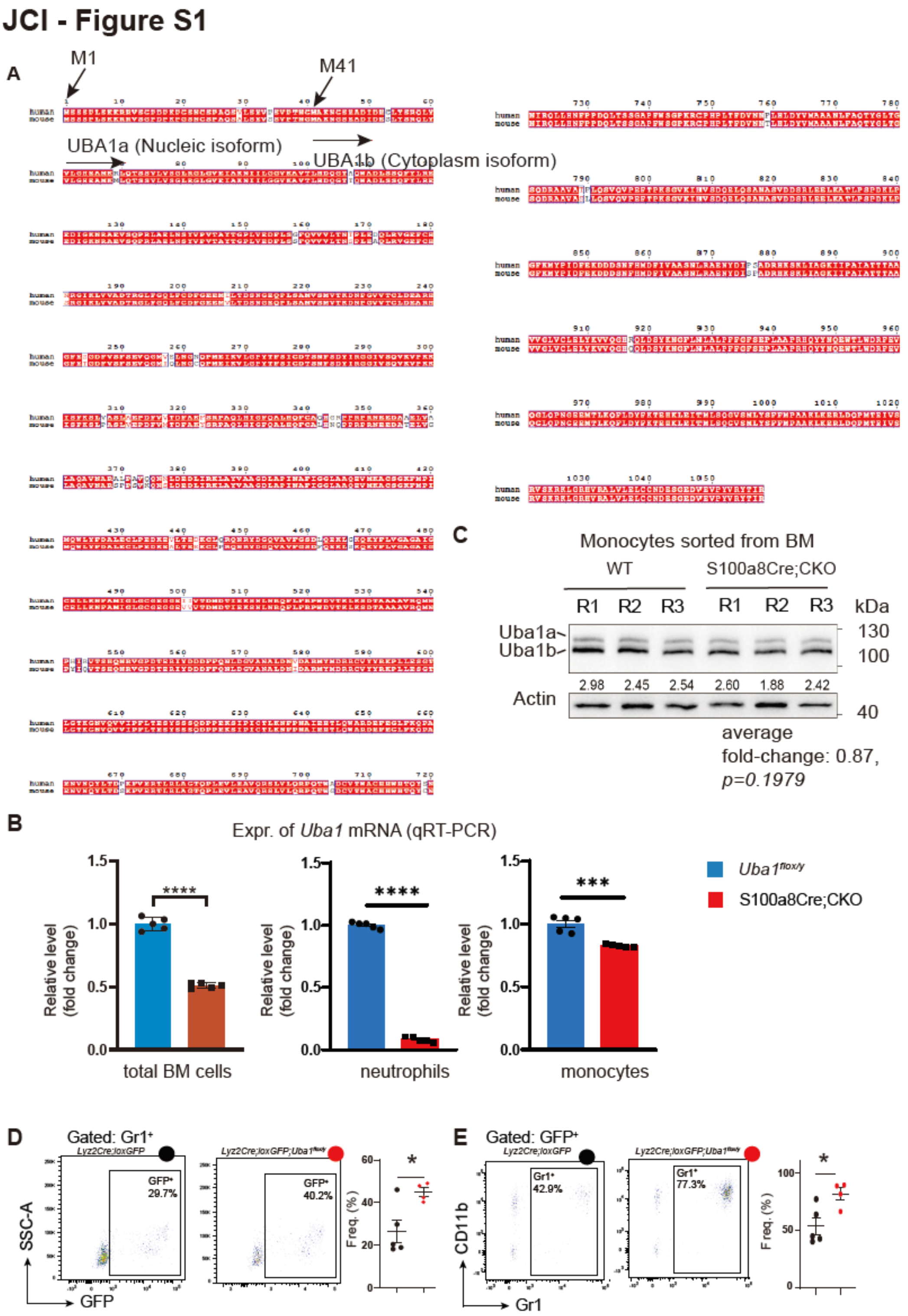

Figure S1: Conservation of UBA1 protein sequences in human and mouse and experimental assays on depletion efficiency of Uba1 in the CKO models. Related to **Figure 1** and **4** in the **Main text**.

(A) Protein sequences of human UBA1 and mouse Uba1 are compared. Mouse and human express UBA1 in same length of BM cells (1058 aa) with >95% sequence

identity. The start point of UBA1 isoform is at M1 while that of UBA1b isoform is at M41. Related to **Figure 1.**

(B) Quantification of *Uba1* mRNA expression in total BM cells, neutrophils and monocytes of S100a8Cre;CKO by qRT-PCR. Box plots show the relative expression level of *Uba1* in total BM cells, purified neutrophils and monocytes. Related to **Figure 1.**

(C) Quantification of Uba1 protein expression in monocytes of S100a8Cre;CKO by western blotting. Related to **Figure 1.**

(D) *Lyz2Cre; lox-GFP* and *Lyz2Cre; lox-GFP; Uba1$^{flox/y}$* mice were generated and used for confirmation of *Lyz2Cre* activity in the study. We crossed the *Lyz2Cre; Uba1$^{flox/y}$* CKO mutants with tomato-lox-GFP line to generate *Lyz2Cre; lox-GFP* and *Lyz2Cre; lox-GFP; Uba1$^{flox/y}$*. Expression of GFP indicates the activity of *LyzCre* in the Cre/loxP strategy. Related to **Figure 4.**

(E) When gated with Gr1^{+} channel or with GFP^{+} channel, we confirmed that *Lyz2Cre* activity is robust (an indirect indication for loss of *Uba1*) in Gr1^{+} myeloid cells in *Lyz2Cre; lox-GFP; Uba1$^{flox/y}$*. Interestingly such loss of *Uba1* in *Lyz2Cre; lox-GFP; Uba1$^{flox/y}$* did not induce death in Gr1^{+} myeloid cells. In contrast, mutant Gr1^{+} myeloid cells appear to have longer life-span than that WT Gr1^{+} myeloid cells. Related to **Figure 4.**

ns, not significant; *, p<0.05; **, p<0.01; ***, p<0.001; ****, p<0.0001; n=3~5 biological repeats.

JCI - Figure S2

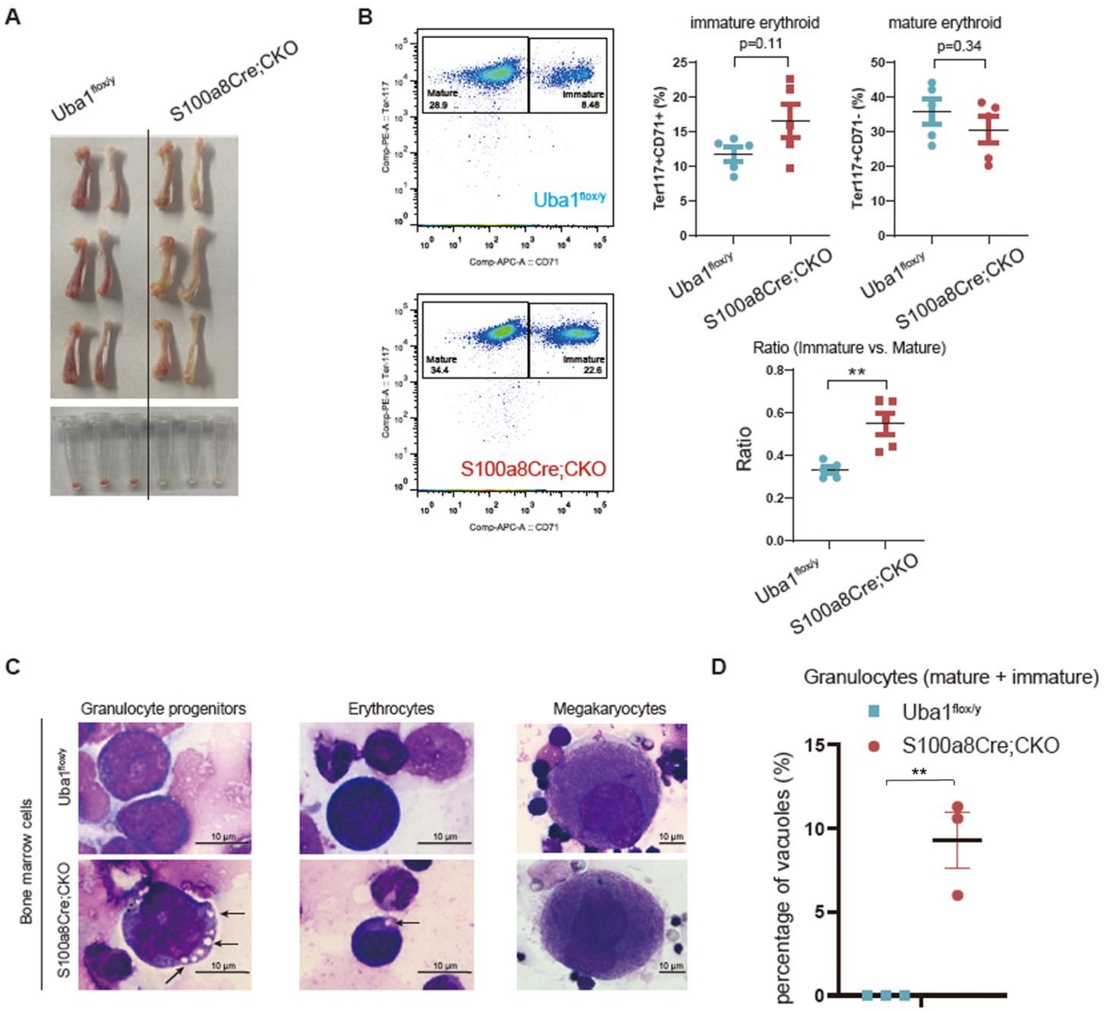

Figure S2 Experimental analysis of erythropoiesis and vacuolization in the BM cells. Related to **Figure 5** in the **Main text**.

(**A-B**) Slightly impaired erythropoiesis in the bone marrow of S100a8Cre;CKO mice. **A**, visual examination of the pellet when purifying the BM cells. Representative flow cytometry profile of erythropoiesis in the BM (**B**, left panel). Significant changes were determined only when calculating the ratio of immature erythrocytes against mature erythrocytes (**B**, right panel).

(**C-D**) Giemsa staining of mouse BM smears and quantification of vacuolization in granulocytes.

ns, not significant; *, p<0.05; **, p<0.01; ***, p<0.001; ****, p<0.0001; n=3~5 biological repeats.

JCI - Figure S3

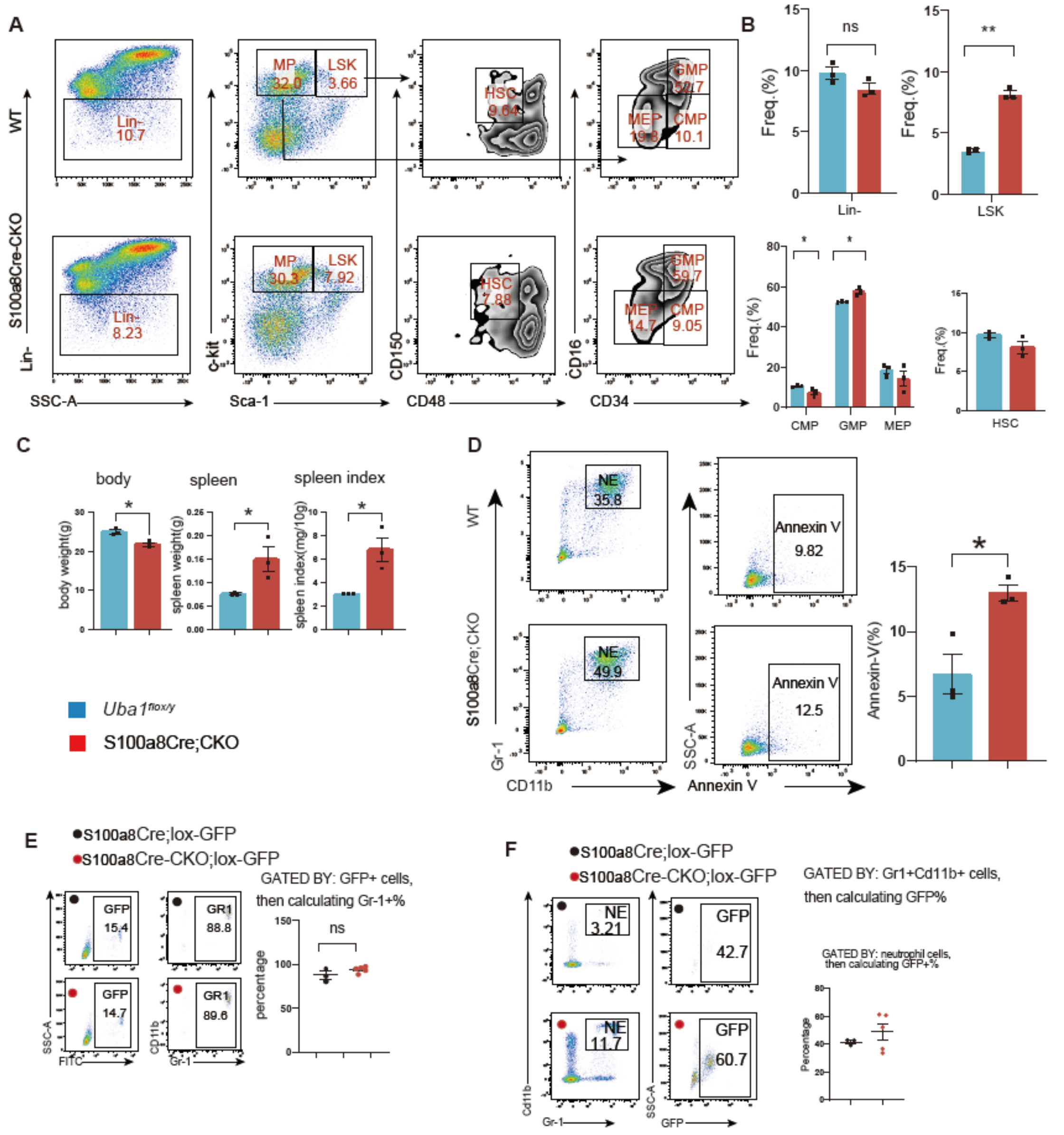

Figure S3: Flow cytometry analysis on bone marrow cells from *S100a8Cre-CKO* mice. Related to **Figure 5 and 6** in the **Main text**.

Potential hematopoietic abnormalities were examined by flow cytometry using bone marrow (BM) or peripheral blood cells. BM or PB cells from at least 3 WT animals and 3 S100a8Cre-CKO animals for the flow cytometry examination.

(A) Gating strategies and representative flow cytometry profiles for hematopoiesis using bone marrow cells from WT and *S100a8Cre-CKO* mice.

(B) Quantification of various compartment of hematopoietic progenitor cells as indicated.

(C) Body weight, spleen weight and spleen index in the WT and S100a8Cre;CKO mice.

(D) Apoptosis analysis of neutrophils in the bone marrow. Left panel, Gating strategies and representative flow cytometry profile; Right panel, Quantification of apoptotic cells (Annexin-V^{+}) in neutrophils.

(E-F) *S100a8Cre-CKO* mice were bred with lox-GFP mice for validating the activity

of S100a8Cre. **E**, GFP^+ cells were gated and for calculating myeloid cells. **F**, neutrophil cells were gated and for GFP^+ cells.

ns, not significant; *, p<0.05; **, p<0.01.

JCI - Figure S4

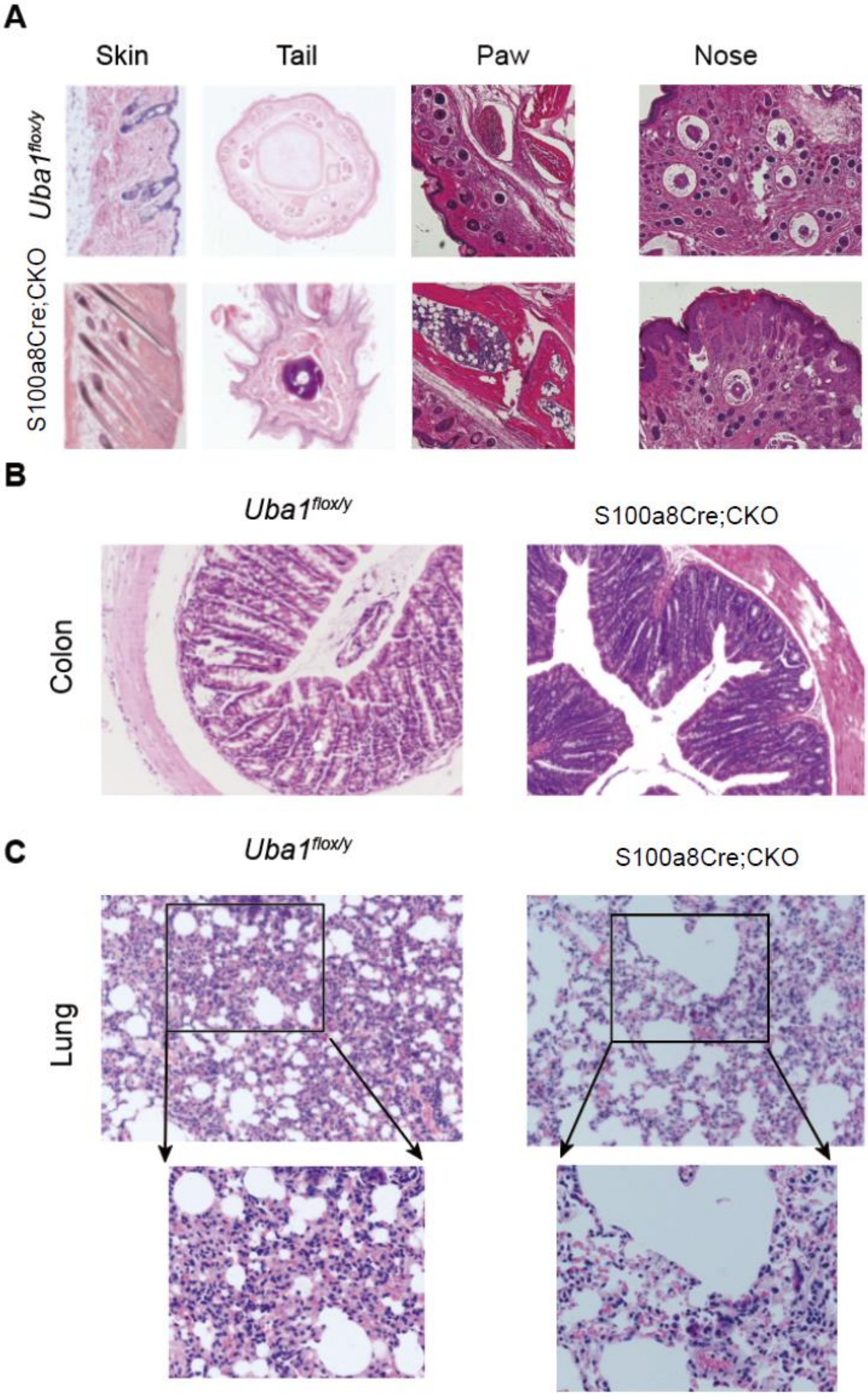

Figure S4: H&E staining of various organs from *S100a8Cre-CKO* mice. Related to **Figure 5** in the **Main text**.

Affected tissues were collected from at least 3 WT animals and 3 S100a8Cre-CKO animals for H&E histology examination.

(A) H&E staining of affected tissues including skin, tail, paw and nose. Note that skin

at the back and tail in *S100a8Cre-CKO* mice is not as smooth as in WT. Infiltration of immune cells are observed in the swollen paws in the *S100a8Cre-CKO* mice. Although flare noses in the *S100a8Cre-CKO* mice were observed, we failed to observe much dramatic difference in the H&E histology.

(B) H&E staining of colon tissues.

(C) H&E staining of lung tissues.

JCI - Figure S5

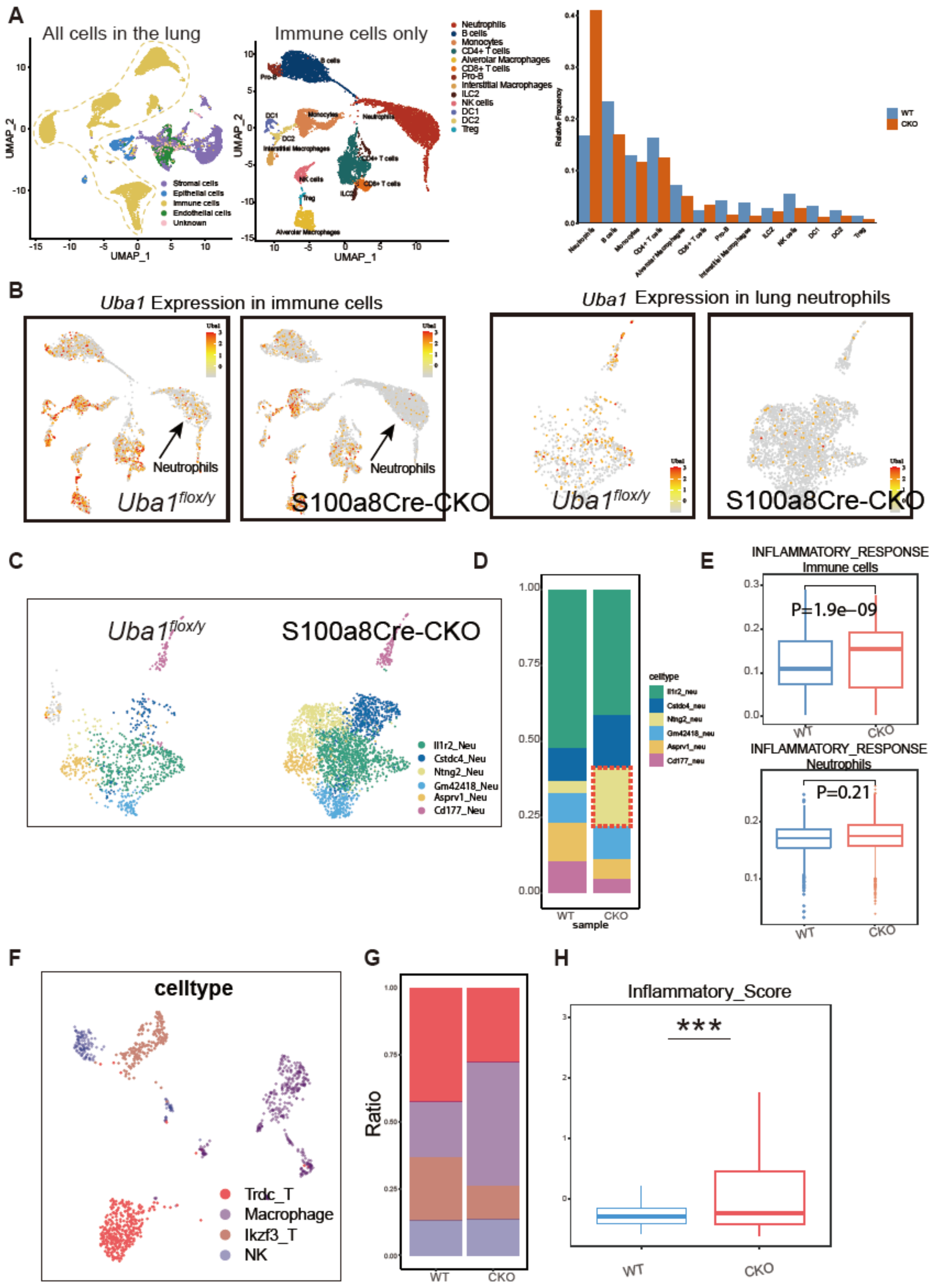

Figure S5: scRNA-seq analysis of lung tissues from *S100a8Cre-CKO* mice. Relying on scRNA-seq dataset, we observed an obvious increased inflammation in the lung tissue from *S100a8Cre-CKO* mice. Tissues were from 1 WT animal and 1 *S100a8Cre-CKO* animal. Related to **Figure 5** in the **Main text**.
(**A**) Lung tissues from WT and *S100a8Cre-CKO* mice were subjected for scRNA-

sequencing analysis for identifying any possible pathophysiological alterations in the *S100a8Cre-CKO* mice. Immune cells were extracted and subject for further analysis. Left panel: the UMAP plot of total cells of the lung tissue; middle panel: the UMAP plot of the immune cells of the lung tissue; right panel: proportions of various immune cell types in the immune cell pool.

(**B**) Expression of *Uba1* in the immune cell pool and in the extracted neutrophils.

(**C**-**D**) Sub-clusters of the neutrophils and their proportions.

(**E**) Inflammatory scoring of the total immune cells or just neutrophils

(**F**-**H**) Analysis of other immune cells except neutrophils by re-clustering(**F**), proportion analysis (**G**) and scoring (**H**).

***, $p<0.001$.

JCI - Figure S6

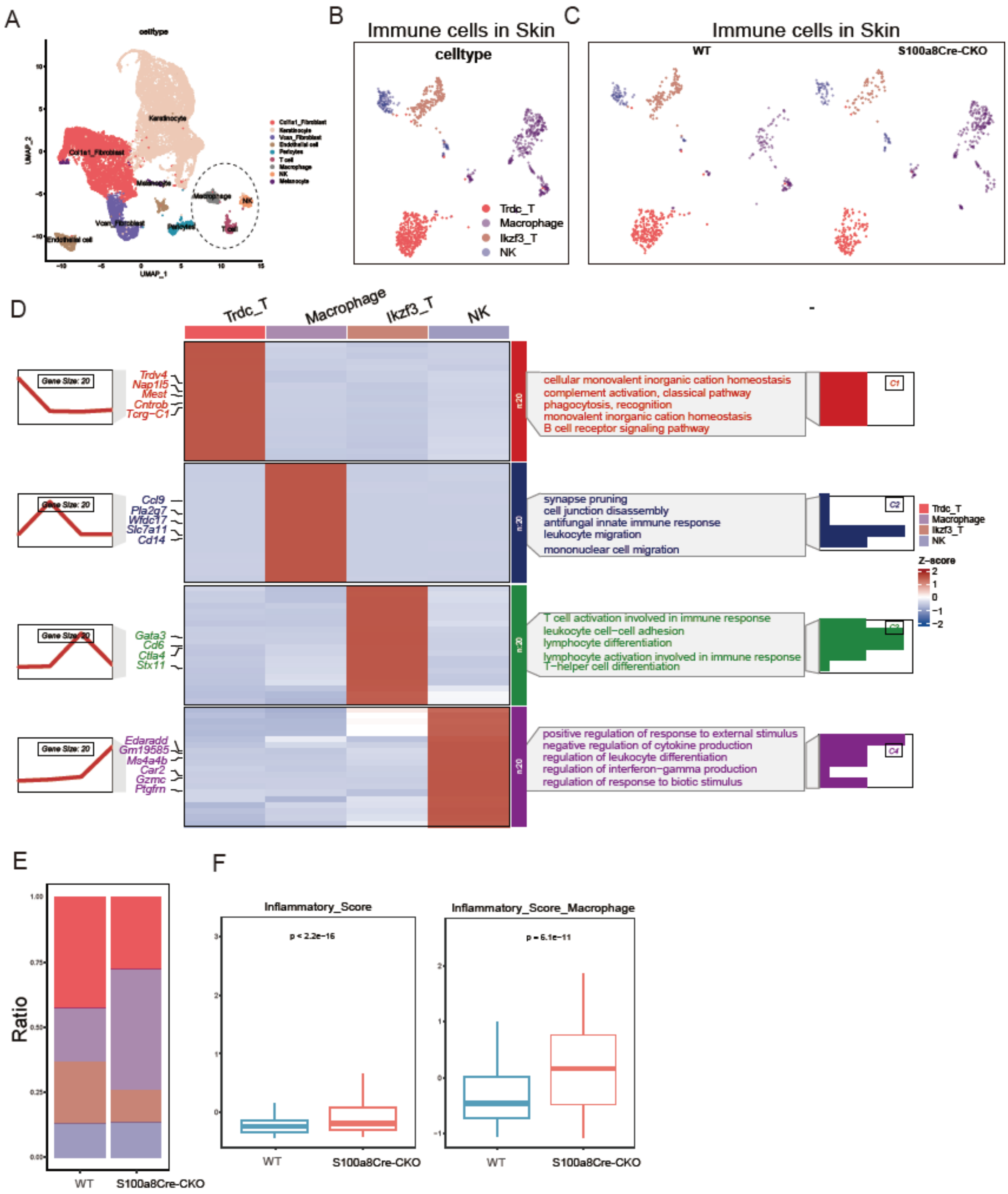

Figure S6: scRNA-seq analysis of skin tissues from *S100a8Cre-CKO* mice. Relying on scRNA-seq dataset, we observed a slightly increased inflammation in the skin tissue from S100a8Cre-CKO mice. Tissues were from 1 WT animal and 1 S100a8Cre-CKO animal. Related to **Figure 5** in the **Main text**.

(**A-C**) Skin tissues from WT and *S100a8Cre-CKO* mice were subjected for scRNA-sequencing analysis for identifying any possible pathophysiological alterations in the *S100a8Cre-CKO* mice. Immune cells were extracted and subject for further analysis. **A**: the UMAP plot of total cells of the skin tissue; **B**: the UMAP plot of the immune cells of the skin tissue; **C**: split plots of skin immune cells. Of note, possibly due to

technical problem, we failed to capture any neutrophils in the skin scRNA-seq.

(**D**) The enriched pathways in each immune cell types form the skin scRNA-seq datasets.

(**E**) Proportions of each immune cell types in the immune cell pool of the skin.

(**F**) Scoring of the inflammatory activity for all immune cells or just macrophages.

JCI - Figure S7

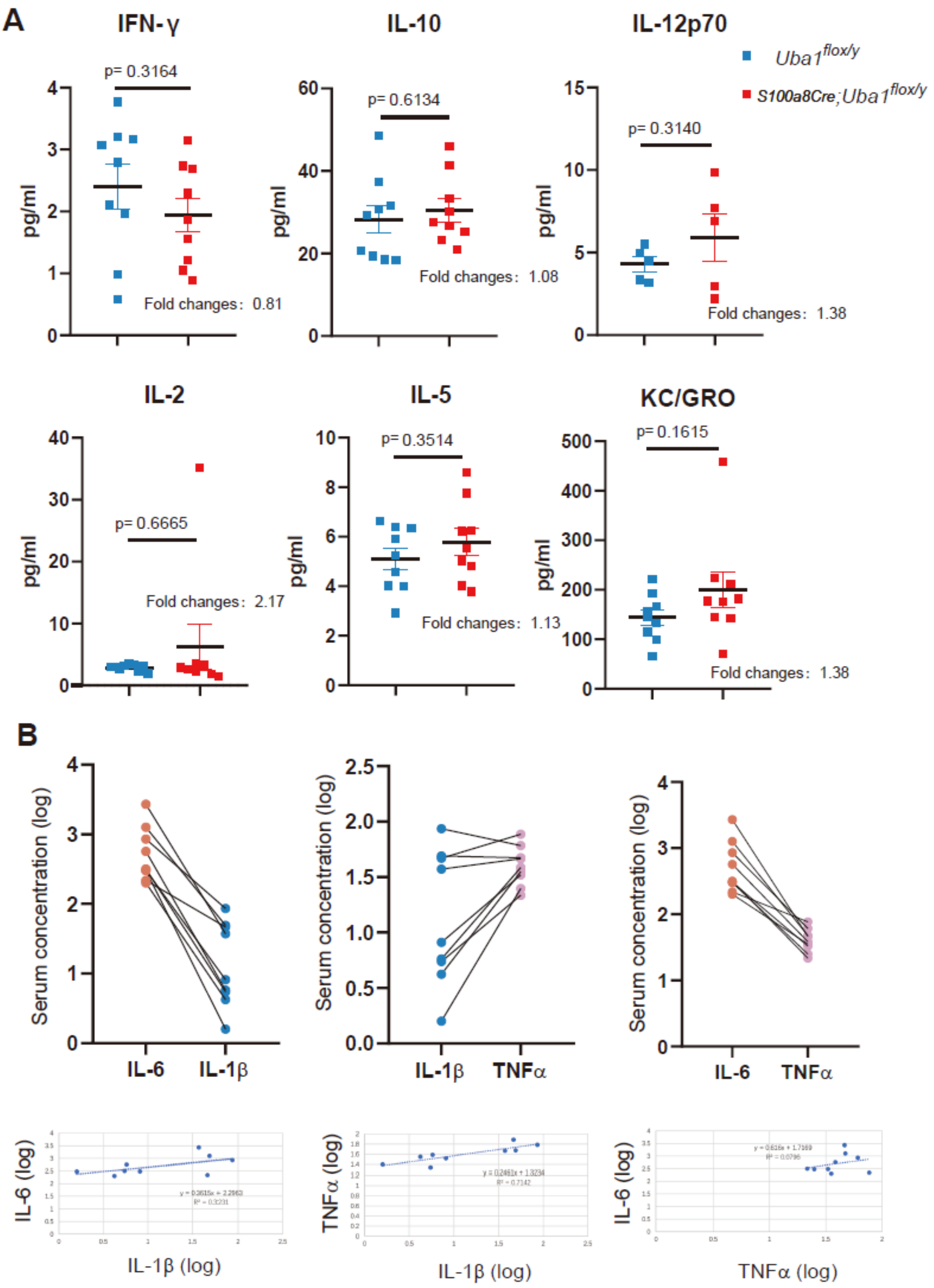

Figure S7: Additional ELISA results of serum cytokines from *S100a8Cre-CKO* mice.

Positive correlation of IL-6, IL-1β and TNFα were observed in the serum of S100a8Cre-CKO mice. Related to **Figure 5H** in the **Main text**.

(A) Serum levels of other 6 cytokines from WT and *S100a8Cre-CKO* mice. Levels of IL-4 are undetectable in wild type and were not shown here.

(B) Correlation analysis of pro-inflammatory cytokines IL-6, IL-1β, and TNFα from *S100a8Cre-CKO* mice.

JCI - Figure S8

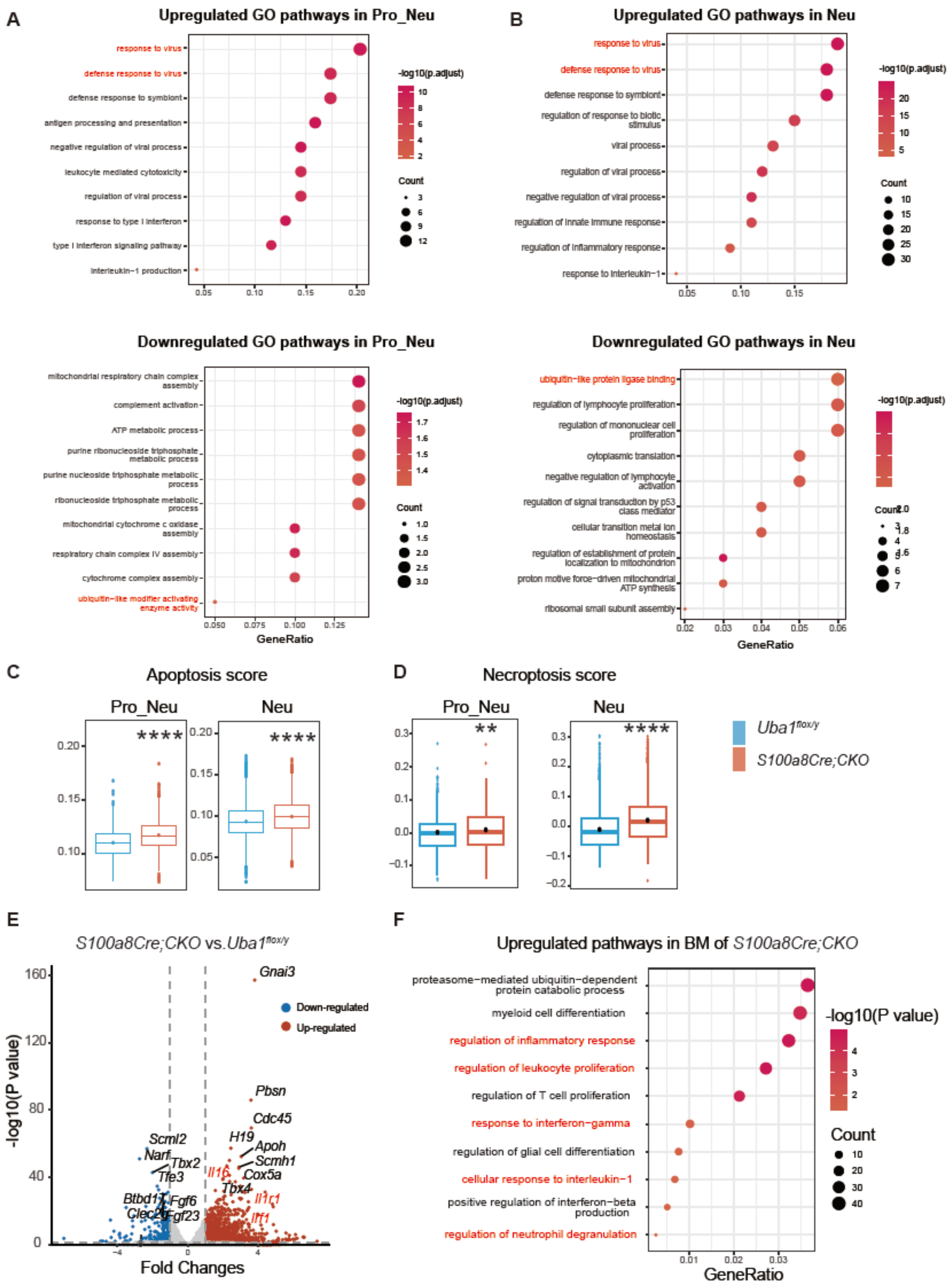

Figure S8: Single-cell and bulk RNA-seq analysis of BM cells, Related to **Figure 6** in the **Main text**.

(A-B) Unbiased GO pathway analysis in Pro_Neu and Neu. Both upregulated pathway and downregulated pathway are presented.

(**C-D**) Loss of *Uba1* in neutrophils results in disturbed cellular homeostasis as indicated by increased Apoptosis Score and Necroptosis Score.

(**E-F**) In parallel with the scRNA-seq analysis, bulk RNA-sequencing analysis was conducted using total BM cells from WT and *S100a8Cre-CKO*. **E**, volcano plot suggesting some pro-inflammatory genes are upregulated in *S100a8Cre-CKO* mice. **F**, enriched pathways in *S100a8Cre;CKO* mice including pro-inflammation response and cell responses to IL-1 and IFN-gamma.

**, $p<0.01$; ****, $p<0.0001$.

JCI - Figure S9

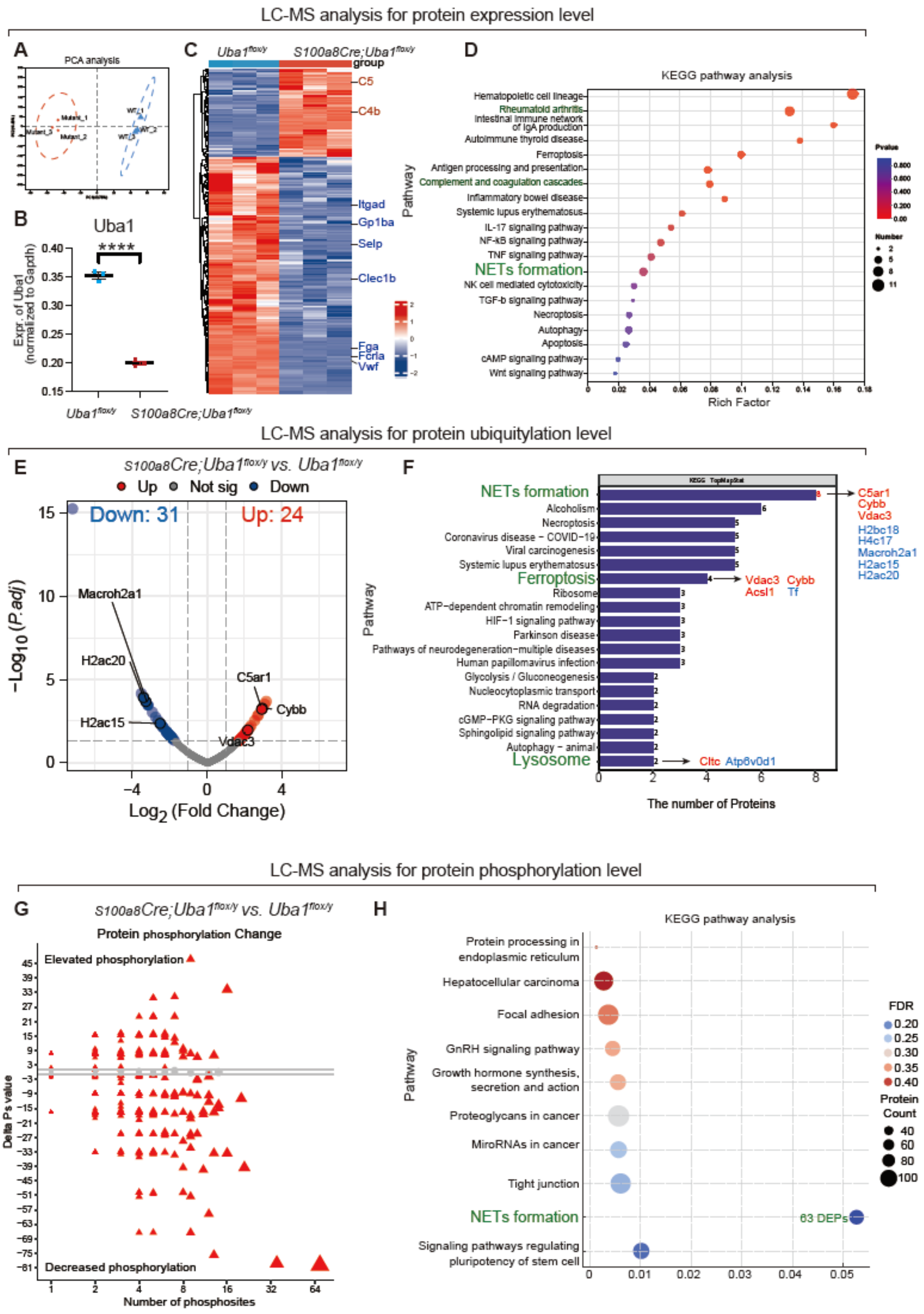

Figure S9: Disturbed neutrophil homeostasis in *S100a8Cre-CKO* revealed by proteomic LC-MS/MS analysis. Related to **Figure 9-10** in the **Main text**.

BM cells (unsorted) from WT and *S100a8Cre-CKO* mice were subject for regular proteomic quantification (**A** to **D**), protein ubiquitylation measurement (**E** to **F**) and protein phosphorylation measurement (**G** to **H**).

(A) PCA analysis of the WT (n=3 biological repeats) and *S100a8Cre-CKO* (n=3

biological repeats) BM cells used for regular protein expression level.

(B) Expression of *Uba1* in the regular protein expression datasets were quantified (normalized to Gapdh). Fold change of Uba1 in unsorted BM cells: ~0.57, p < 0.0001.

(C) Heatmap of 265 different expression proteins (DEPs). In the figure, 2 up-regulated and 7 down-regulated proteins related to NET formation pathway is denoted.

(D) Enriched pathways are highlighted when comparing *S100a8Cre* -CKO BM cells with WT BM cells. Proteins related to rheumatoid arthritis, complement cascade and NETs formation are highlighted.

(E) Volcano plot of different expression level of ubiquitinated proteins. Proteins related to NETs formation are labeled.

(F) Barplot of KEGG enrichment of ubiquitinated DEPs. Lysosome, NETs formation or Ferroptosis-related pathways are enriched when comparing *S100a8Cre-CKO* BM cells with WT BM cells.

(G) Scatter plot of protein phosphorylation status values. Changes of protein phosphorylation levels (ΔPs) was calculated by summing the log2FC values of all differentially phosphorylated peptides corresponding to the same protein. ΔP value >1 or < -1 indicated that the phosphorylated peptide in the protein was up-regulated or down-regulated, represented by red triangles. -1<ΔPvalue<1 indicates that the phosphorylation in this protein has not changed, represented by grey triangles. The size of triangle means number of phosphosites.

(H) Enriched NETs formation-related pathways are highlighted when comparing *S100a8Cre* -CKO BM cells with WT BM cells. In total 63 phosphorylated DEPs were enriched in NET formation pathway.

****, p<0.0001; n=3~4 biological repeats.

JCI - Figure S10

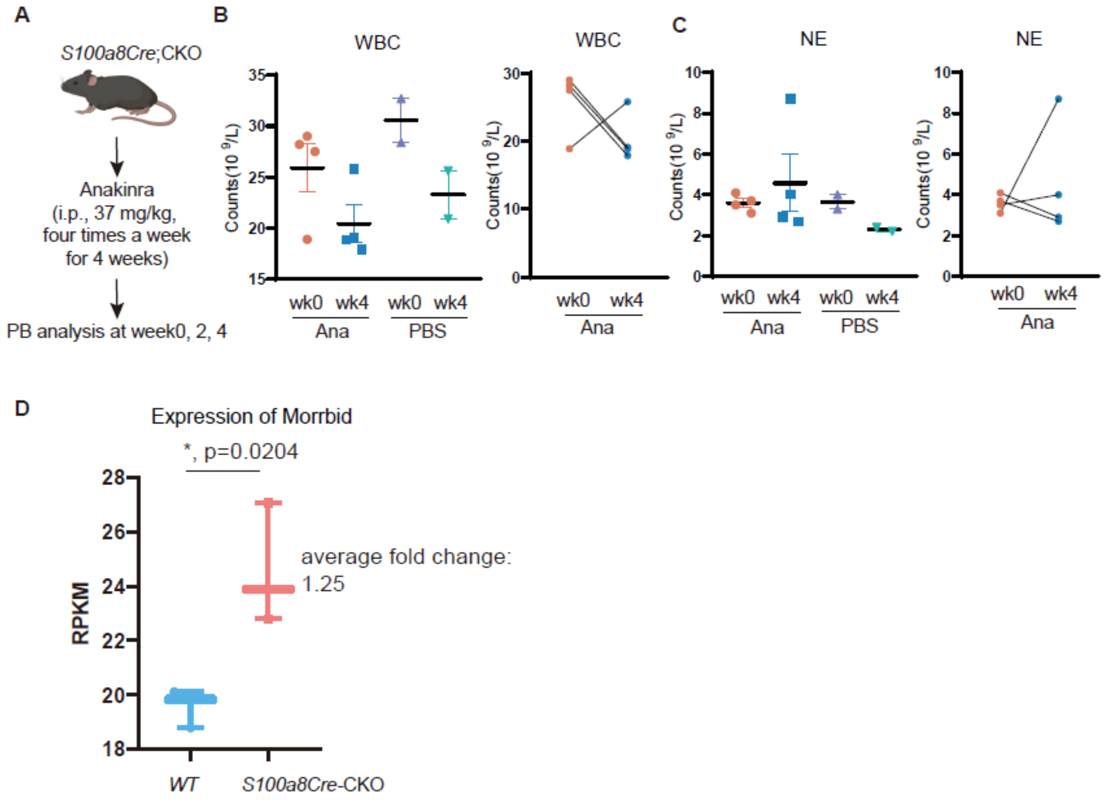

Figure S10: Additional results of drug treatment on the *S100a8Cre-CKO* mice. Treatment with Anakinra partially reversed abnormalities in *S100a8Cre-CKO* mice. Related to **Figure 11-12** in the **Main text**.

(A) Scheme of the regime for the Anakinra treatment on *S100a8Cre* -CKO mice.

(B) Counts of WBC at the time-points pre or post Anakinra treatment.

(C) Counts of neutrophils at the time-points pre or post Anakinra treatment.

(D) Expression of *Morrbid* in bone marrow cells of WT and *S100a8Cre-CKO* mice. Bulk RNA-seq dataset was normalized to RPKM for each gene.

*, $p<0.05$; n=3~4 biological repeats.
