# Supplementary material for: UBA1-depleted neutrophils disrupt immune homeostasis and induce VEXAS-like autoinflammatory disease in mice
Source: J Clin Invest. 2025 Sep 4;135(21):e193011. doi: 10.1172/JCI193011 (PMC12578396; doi:10.1172/JCI193011)

Figure 1D

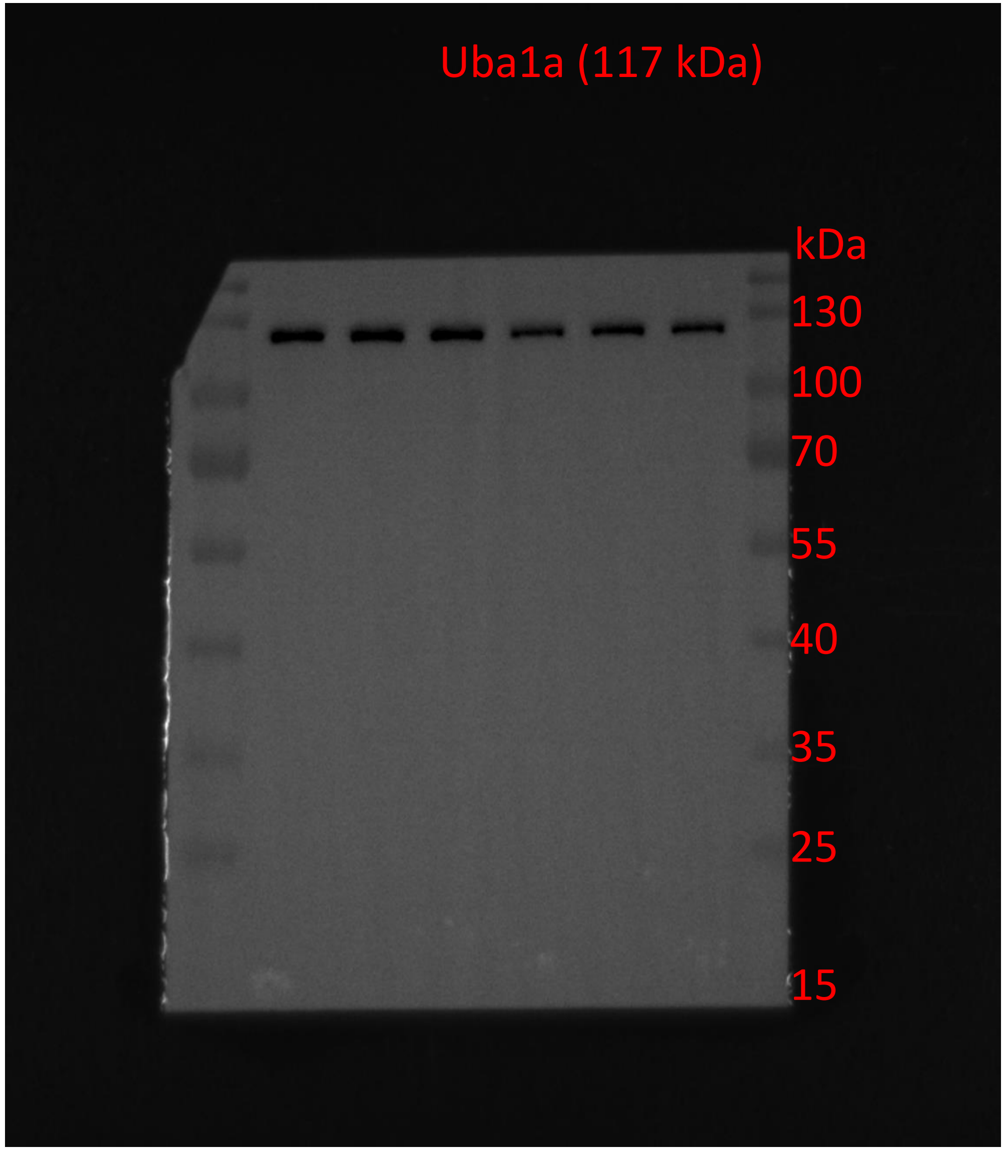
Uba1a (117 kDa)
kDa
130
100
70
55
40
35
25
15

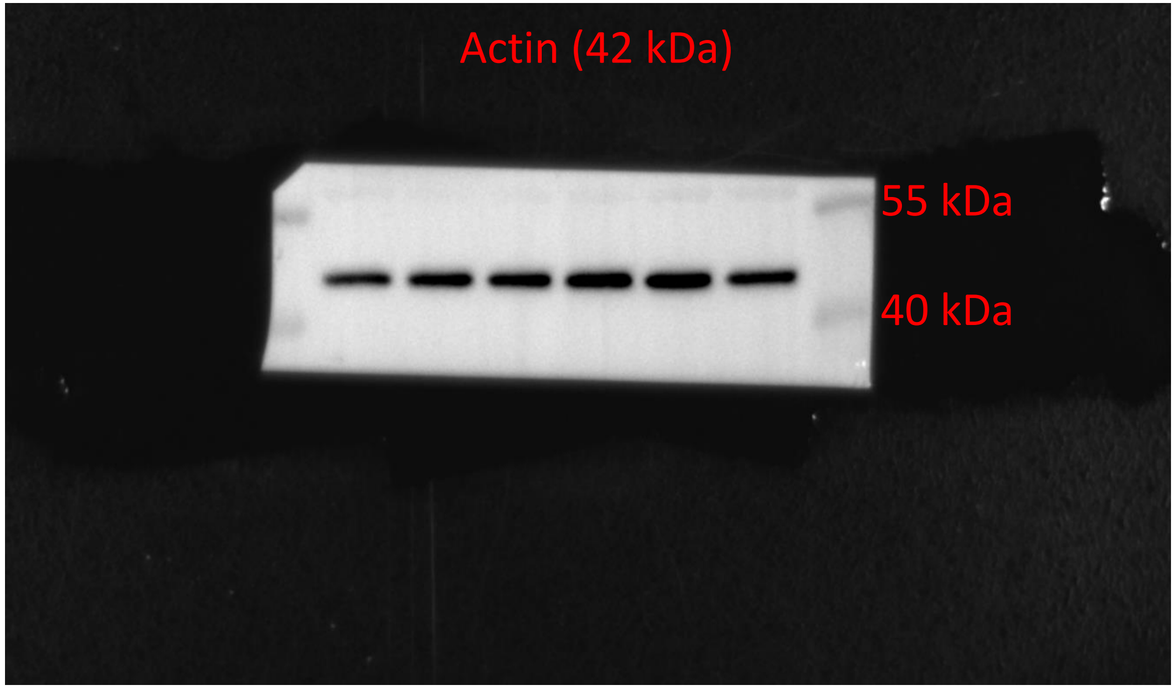
Actin (42 kDa)
55 kDa
40 kDa

Figure 1E

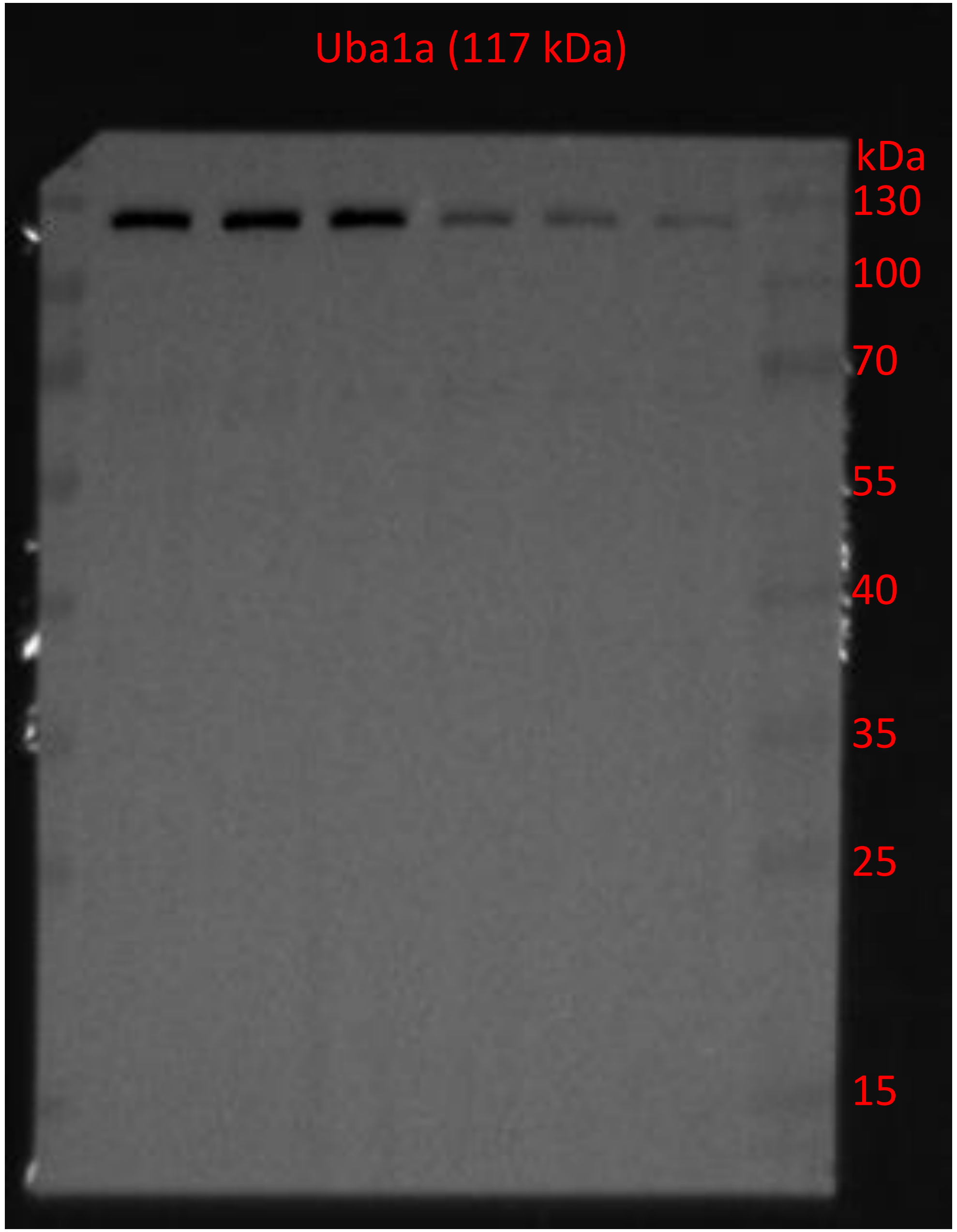

Uba1a (117 kDa)
kDa
130
100
70
55
40
35
25
15

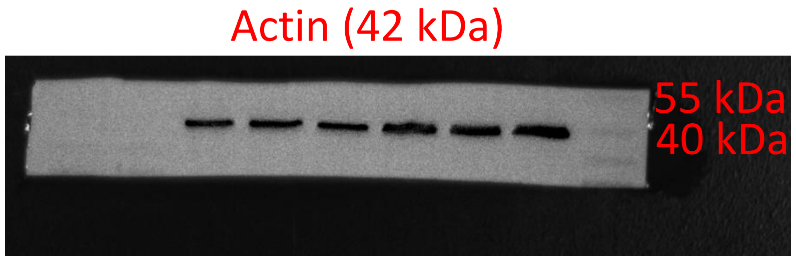

Actin (42 kDa)
55 kDa
40 kDa

Figure 1F

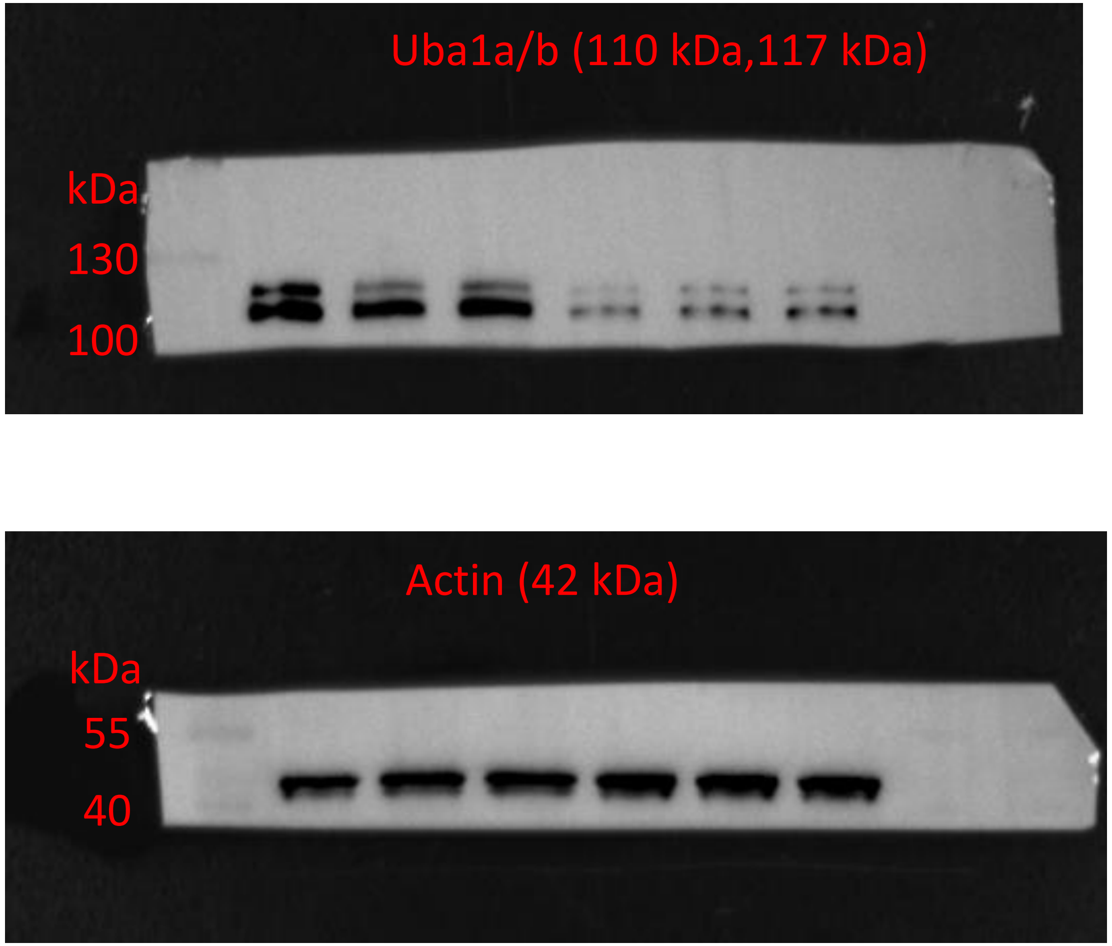

Figure 1G

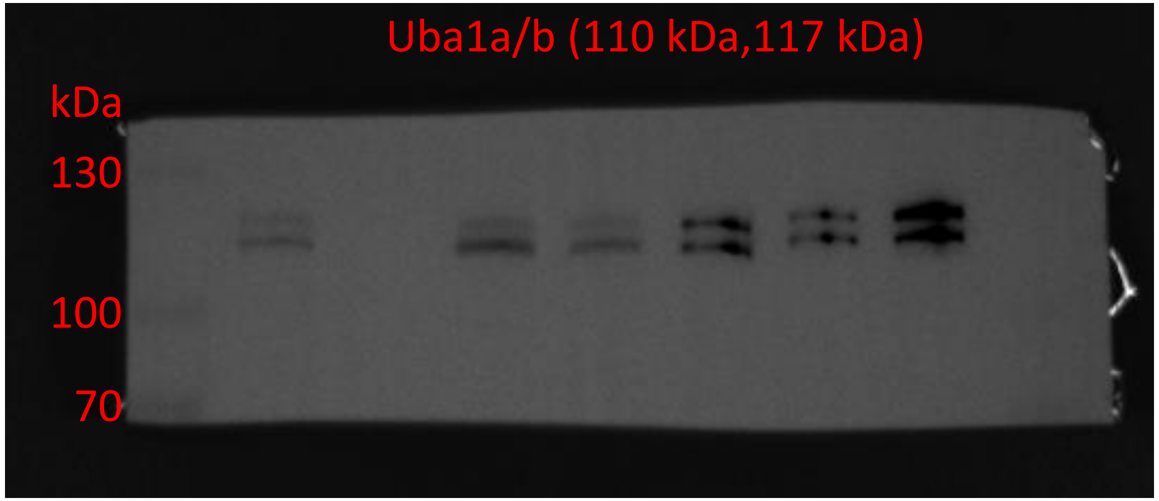

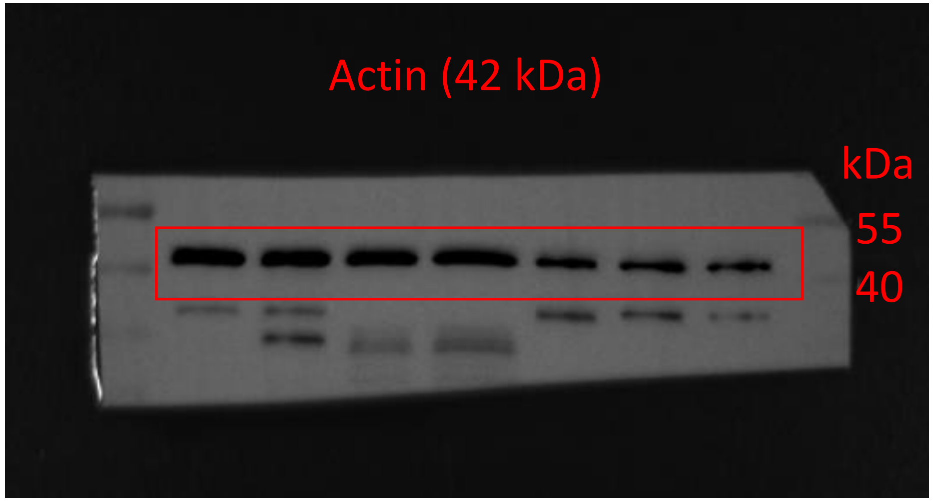

Figure 4F

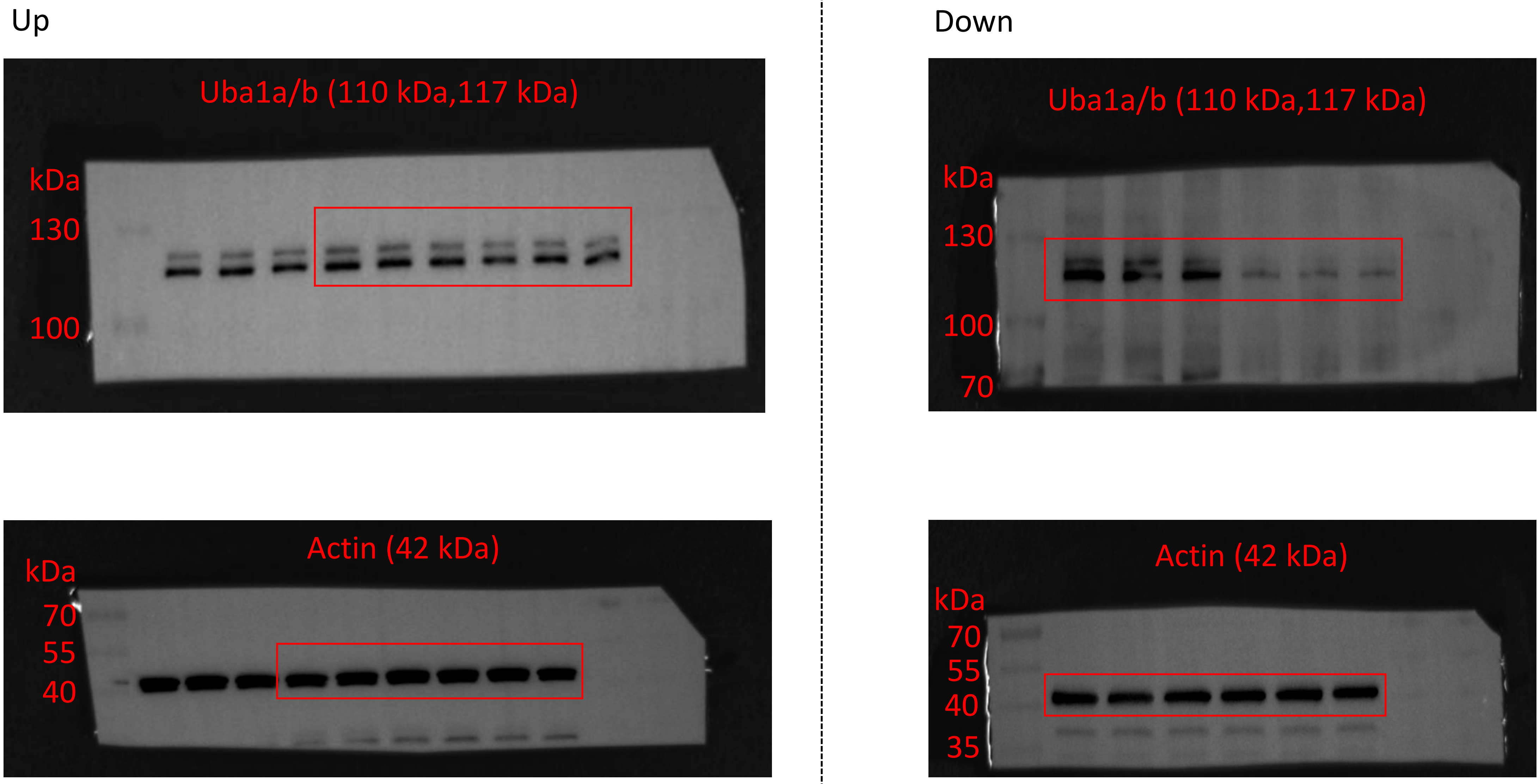
Up
Uba1a/b (110 kDa,117 kDa)
kDa
130
100
Actin (42 kDa)
kDa
70
55
40
Down
Uba1a/b (110 kDa,117 kDa)
kDa
130
100
70
Actin (42 kDa)
kDa
70
55
40
35

Figure 6A

Up

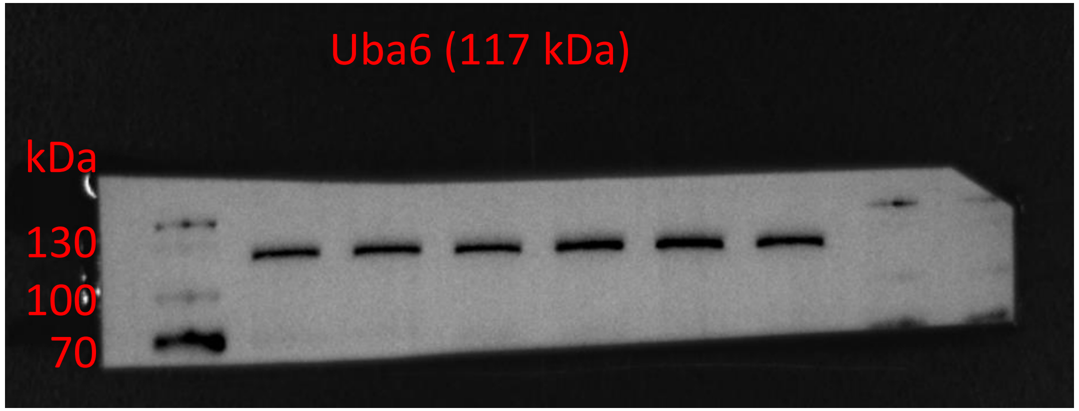

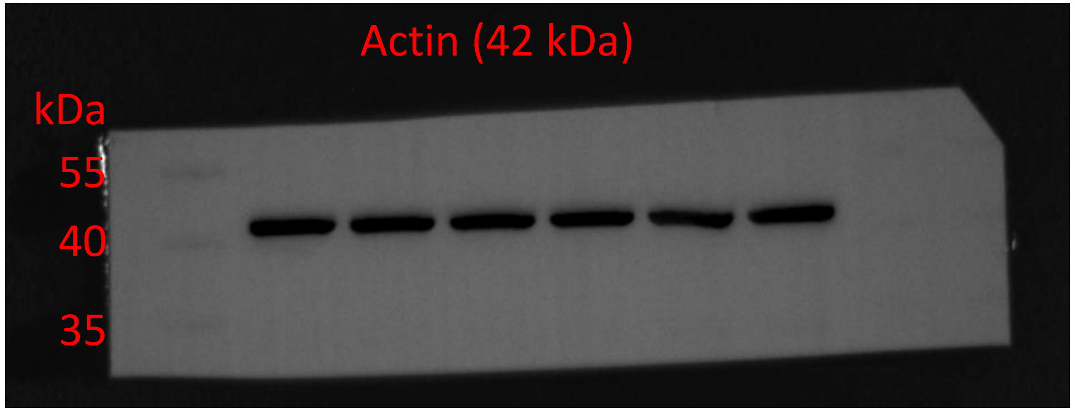

Down

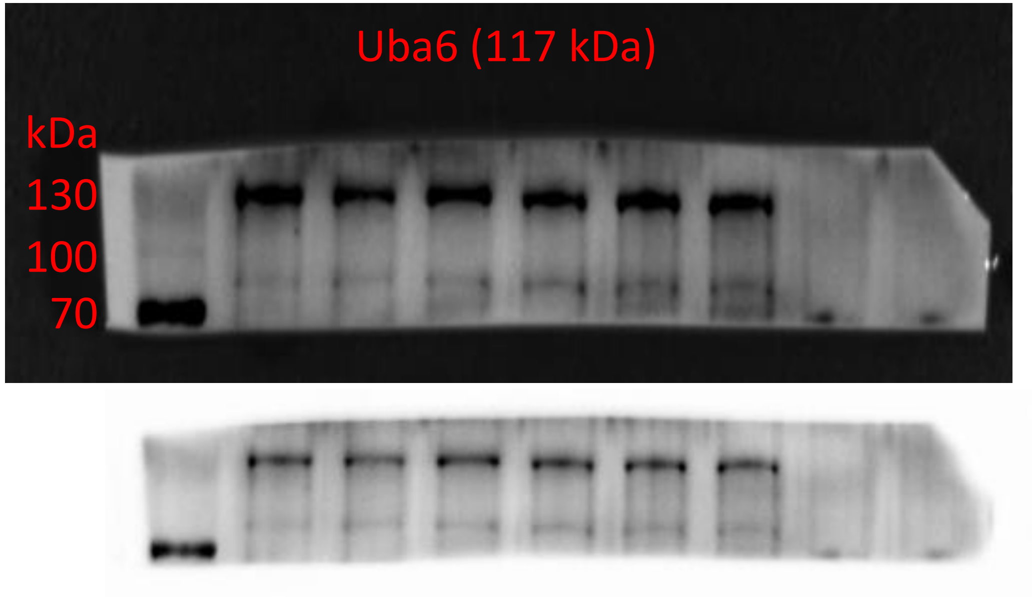

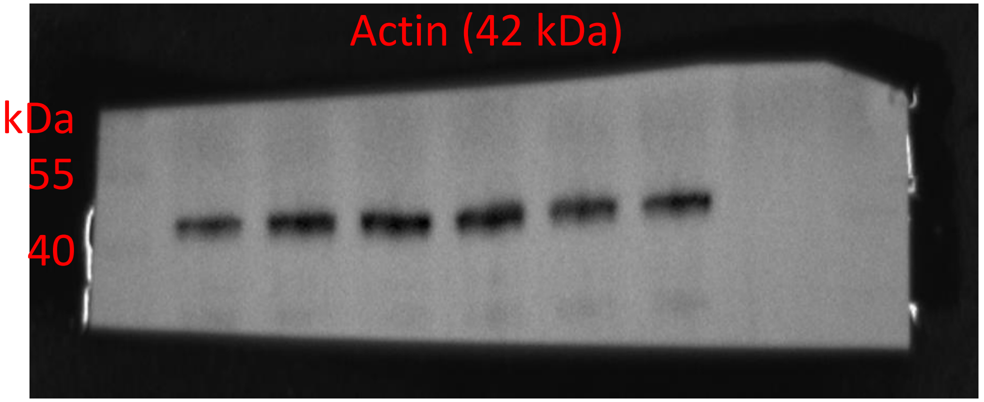

Figure 6B

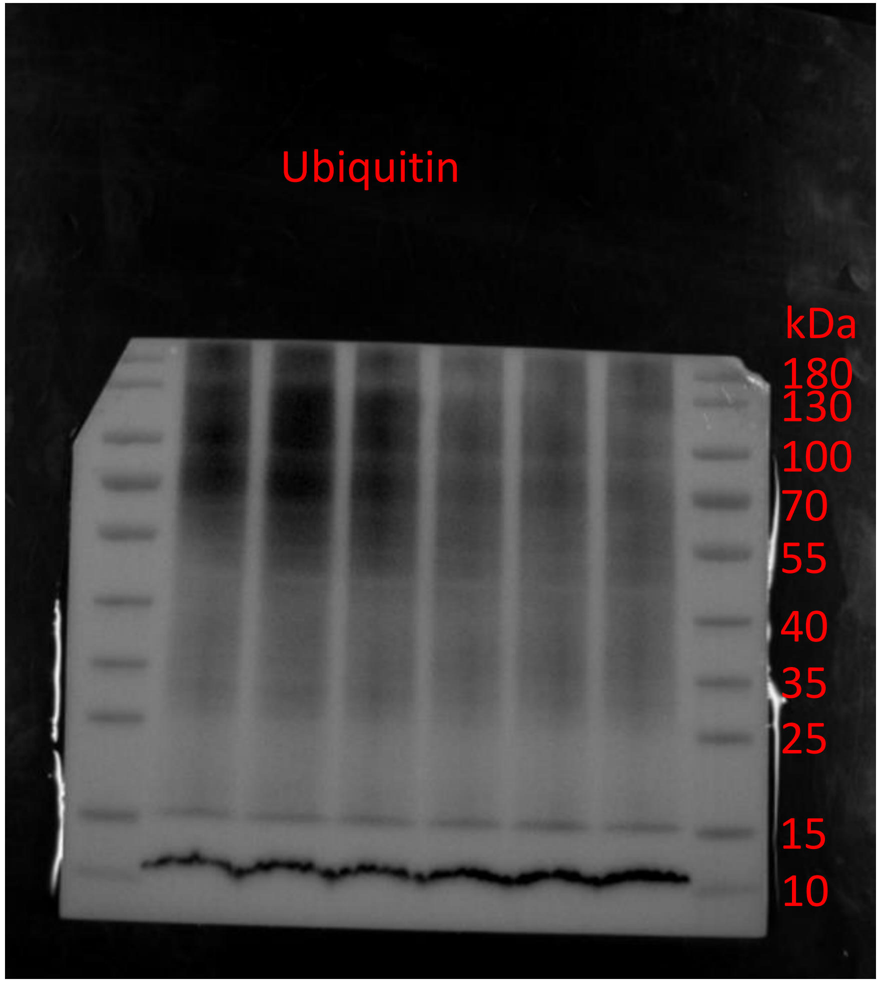
Ubiquitin
kDa
180
130
100
70
55
40
35
25
15
10

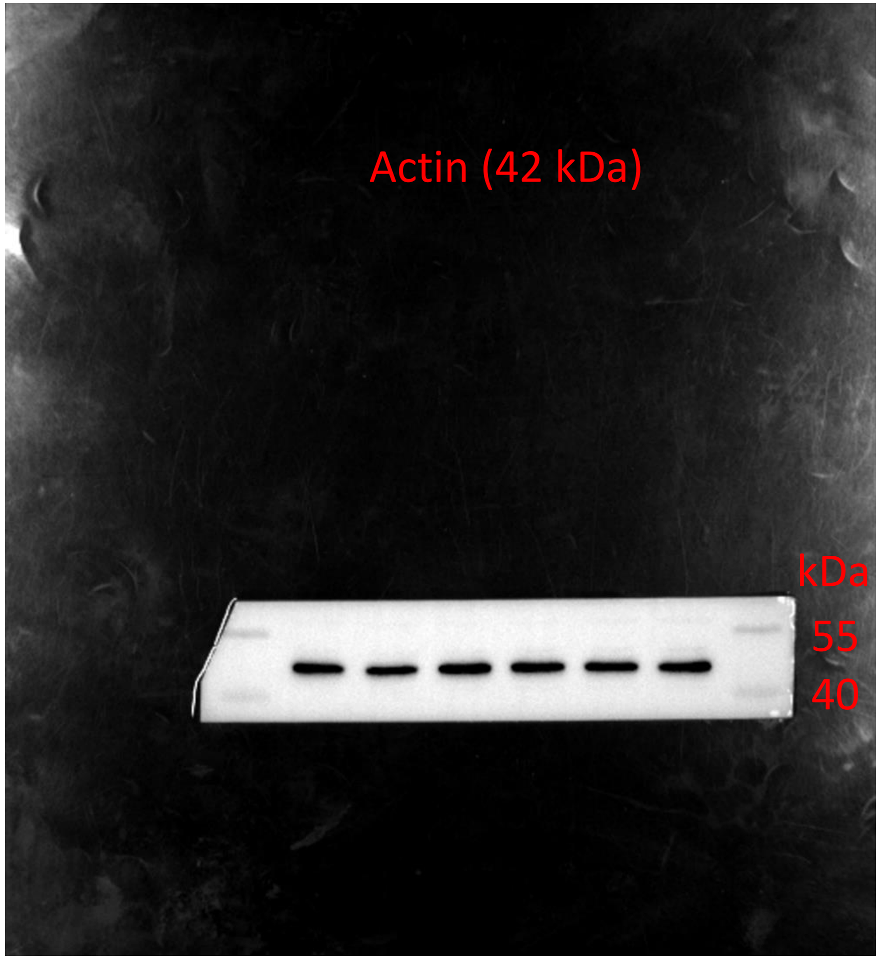
Actin (42 kDa)
kDa
55
40

Figure 7B

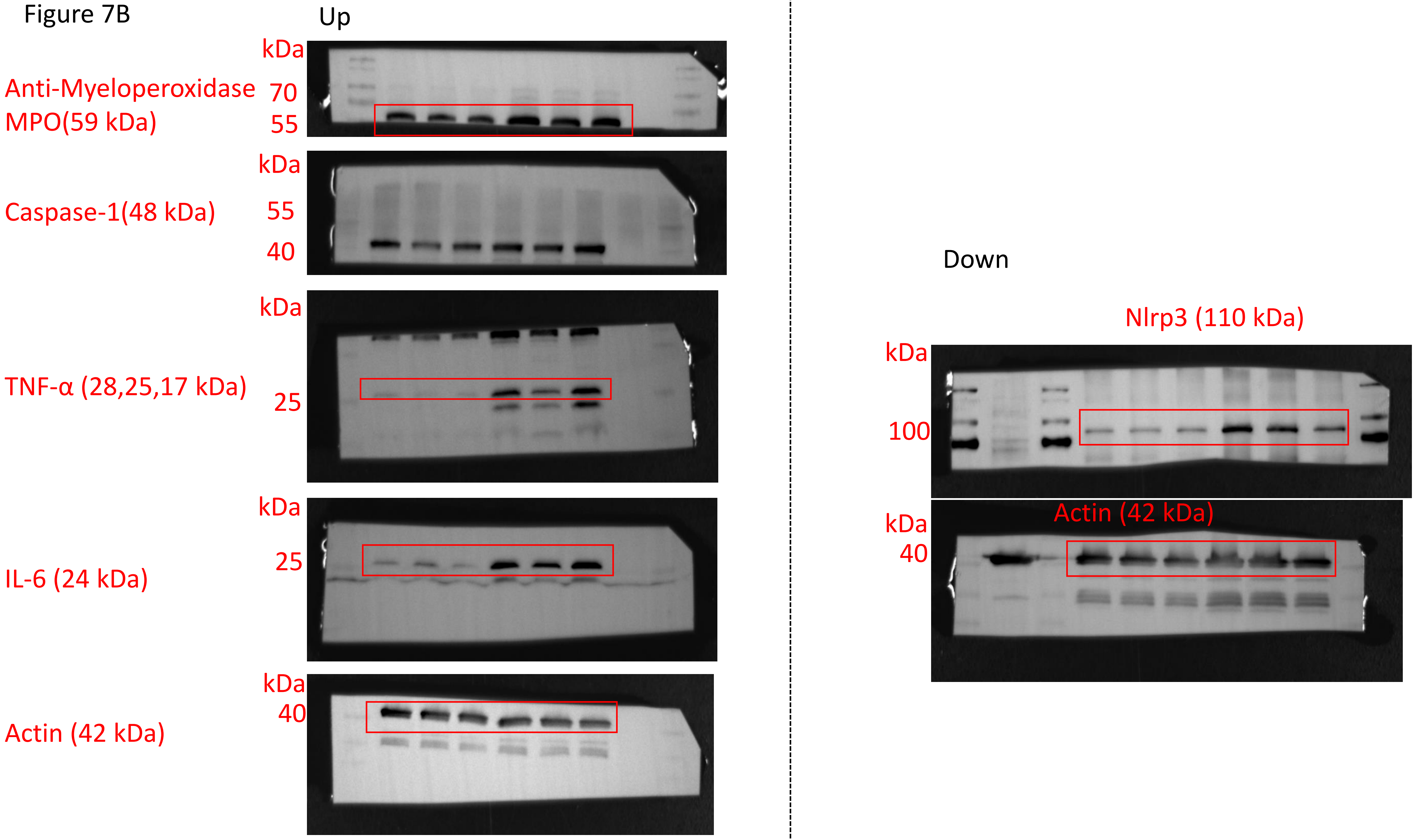
Up
kDa
Anti-Myeloperoxidase
MPO(59 kDa)
70
55
kDa
Caspase-1(48 kDa)
55
40
kDa
TNF-α (28,25,17 kDa)
25
kDa
IL-6 (24 kDa)
25
kDa
Actin (42 kDa)
40
Down
Nlrp3 (110 kDa)
kDa
100
Actin (42 kDa)
kDa
40

Figure S1C

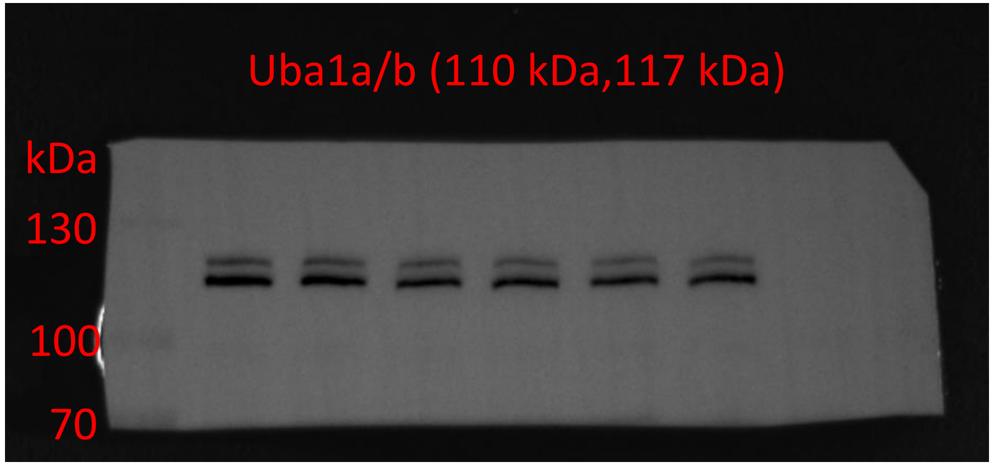

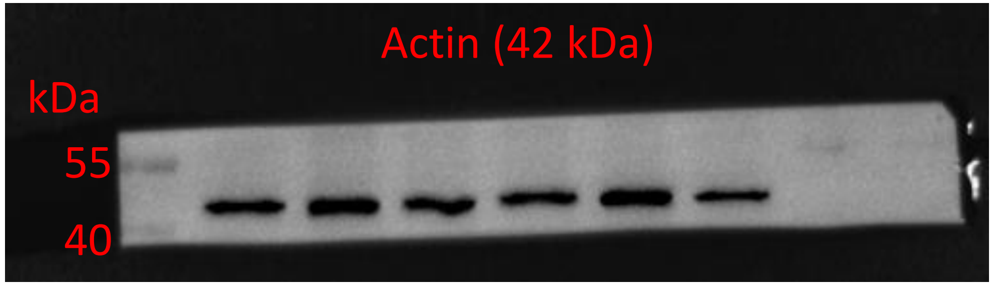

Supplement: Unedited blot and gel images [file jci-135-193011-s033.pdf]
